# Effects of *In Utero* PFOS Exposure on Epigenetics and Metabolism in Mouse Fetal Livers

**DOI:** 10.1021/acs.est.3c05207

**Published:** 2023-09-27

**Authors:** Tsz Chun Ho, Hin Ting Wan, Wang Ka Lee, Thomas Ka Yam Lam, Xiao Lin, Ting Fung Chan, Keng Po Lai, Chris Kong Chu Wong

**Affiliations:** † Croucher Institute for Environmental Sciences, Department of Biology, Hong Kong Baptist University, Kowloon 999077, Hong Kong SAR, China; ‡ State Key Laboratory in Environmental and Biological Analysis, Hong Kong Baptist University, Kowloon 999077, Hong Kong SAR, China; § Department of Psychiatry, Icahn School of Medicine at Mount Sinai, New York, New York 10029, United States; ∥ School of Life Sciences, State Key Laboratory of Agrobiotechnology, Bioinformatics Centre, The Chinese University of Hong Kong, New Territories 999077, Hong Kong SAR, China; ⊥ Key Laboratory of Environmental Pollution and Integrative Omics, Education Department of Guangxi Zhuang Autonomous Region, Guilin Medical University, Guilin 541100, China

**Keywords:** MS-imaging, whole-genome bisulfite sequencing, hepatokine, PPAR, AMPK, ChREBP, MIHA

## Abstract

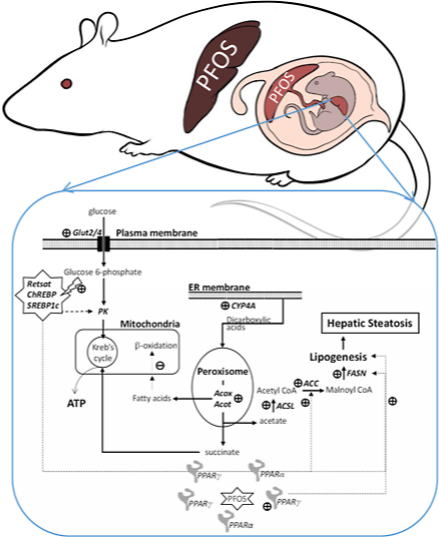

Prenatal exposure to perfluorooctanesulfonate (PFOS) increases fetus’ metabolic risk; however, the investigation of the underlying mechanism is limited. In this study, pregnant mice in the gestational days (GD, 4.5–17.5) were exposed to PFOS (0.3 and 3 μg/g of body weight). At GD 17.5, PFOS perturbed maternal lipid metabolism and upregulated metabolism-regulating hepatokines (*Angptl4, Angptl8, and Selenop*). Mass-spectrometry imaging and whole-genome bisulfite sequencing revealed, respectively, selective PFOS localization and deregulation of gene methylation in fetal livers, involved in inflammation, glucose, and fatty acid metabolism. PCR and Western blot analysis of lipid-laden fetal livers showed activation of AMPK signaling, accompanied by significant increases in the expression of glucose transporters (*Glut2/4*), hexose-phosphate sensors (*Retsat* and *ChREBP*), and the key glycolytic enzyme, pyruvate kinase (*Pk*) for glucose catabolism. Additionally, PFOS modulated the expression levels of PPARα and PPARγ downstream target genes, which simultaneously stimulated fatty acid oxidation (*Cyp4a14, Acot*, and *Acox*) and lipogenesis (*Srebp1c*, *Acaca*, and *Fasn*). Using human normal hepatocyte (MIHA) cells, the underlying mechanism of PFOS-elicited nuclear translocation of ChREBP, associated with a fatty acid synthesizing pathway, was revealed. Our finding implies that *in utero* PFOS exposure altered the epigenetic landscape associated with dysregulation of fetal liver metabolism, predisposing postnatal susceptibility to metabolic challenges.

## Abstract 2

Low-dose PFOS exposure during gestation alters fetal epigenetics and energy metabolism, resulting in long-term health effects.

## Introduction

Environmental chemicals are a nontraditional risk factor for metabolic diseases. Most studies have been conducted on organic pollutants, including pesticides, dioxins, and industrial chemicals such as polychlorinated biphenyls, polybrominated diphenyl ethers, and perfluoroalkyl substances (PFASs), to underpin their metabolic-disrupting effects.^1,2^ Among those chemicals, PFASs are coined as “forever chemicals” that contaminate public water systems and linger in the environment, wildlife, and humans.^3^ In spite of the first lawsuit against PFAS contamination in 1999 and the Stockholm Convention listing of PFOS and PFOA in 2009, human exposure to these chemicals continues to be a major health concern.^4,5^ In fact, the hazardous effects of PFASs on human health are far reaching than we originally thought. PFAS-induced metabolic abnormalities, including dyslipidemia, insulin resistance, and nonalcoholic fatty liver disease, were resulted from their complex biological actions through both peroxisome proliferator-activated receptors (PPAR)-dependent and -independent fatty acid mimicry pathways that involve multiple tissues and signaling processes.^6–8^ PPARs regulate diverse downstream gene targets involved in glucose and lipid metabolism. In addition, PFAS interactions with fatty acid-binding proteins, fatty acid transporters, and metabolic enzymes could disrupt multiple metabolic pathways.^8,9^ Furthermore, a number of fatty acid-responsive nuclear receptors appeared to be affected by PFASs, including constitutive androstane (CAR), farnesoid X (FXR), pregnancy X (PXR), and liver X (LXR).^10–12^

Based on the SWAN (study of women’s health across the nation), serum PFAS concentrations were positively related to diabetes risk.^13^ The Shanghai Birth Cohort study reported similar findings.^14^ Concerns over the toxicity of PFAS were extended to *in utero* exposure in humans. Offspring would be at higher risk of obesity and metabolic disorders^15,16^ due to disruption of the maternal metabolism and placental transfer of PFASs.^4,17,18^ In animal studies, *in utero* exposure to PFOS disrupted maternal and intrauterine metabolism, accompanied by significant decreases in placental nutrient transport and reduced fetal weights.^19–21^ Our previous study demonstrated that maternal exposure to PFOS disturbed offspring energy homeostasis, increased susceptibility to dietary challenges, and caused metabolic disturbances later in life.^21^ Recent studies of the Michigan mother–infant pair’s cohort and Health Outcomes and Measures of the Environment (HOME) showed that gestational PFAS exposure was associated with offspring DNA methylation.^22,23^ Animal and human epidemiological studies have suggested that PFASs alter fetal metabolic programming. However, there is limited experimental research on how *in utero* exposure to PFOS influences prenatal epigenetic programming and *in utero* fetal hepatic metabolism.

This study hypothesized that *in utero* PFOS exposure perturbed maternal metabolism and placental functions, resulting in altered epigenetic landscapes and metabolic programming of offspring. Presumably, underlying perturbing effects on fetal metabolism might result from dysregulation of energy sensors (mTOR/AMPK) and transcriptional factors involved in glucose and lipid metabolism, including PPARs, sterol-response-binding protein-1c (SREBP1c), and carbohydrate-responsive element-binding protein (ChREBP). Consequently, the disturbances in glucose–lipid metabolism regulatory circuitry might lead to metabolic maladaptation. This study examined the effects of *in utero* PFOS exposure on maternal liver metabolic parameters at gestation day 17.5, followed by mass-spectrometry imaging, whole-genome bisulfite sequencing (WGBS), and molecular and biochemical analyses of key metabolic regulators and enzymes in fetal livers. Furthermore, the mechanism underlying PFOS-induced fatty acid synthesis *in utero* was revealed using a human normal liver cell line.

## Materials and Methods

### Animals and Experimental Plan

Mouse CD-1 (ICR) was kept in polypropylene cages with sterilized bedding at 23–24 °C and 12 h of light/dark cycle. A standard mouse diet of LabDiet, 5001 Rodents Diet, and water (in glass bottles) was provided. A guideline and regulation approved by Hong Kong Baptist University’s animal ethics committee (REC/20-21/0234) were followed. In the experimentation, mice were bred, and the following morning was determined to be gestational day (GD) 0.5 when sperm-positive smears were recognized. Pregnant mice were housed individually and divided into three groups (control, low-dose, or high-dose PFOS treatment groups) of 8–9 mice each. The mice were provided with free access to food and water under standard conditions. PFOS (perfluorooctanesulfonate, 98% purity, Sigma-Aldrich) was dissolved in dimethyl sulfoxide (Sigma-Aldrich) before mixing with corn oil. In the control group, corn oil was given. The exposed groups received either 0.3 or 3 μg/g of body weight (bw)/day of PFOS in corn oil by oral gavage from GD 4.5 to 17.5. Based on our previous exposure study, the low-dose is equivalent to occupational exposure.^21^ On GD 17.5, cervical dislocations were performed on pregnant mice. As part of the study, fetal body weight and liver weight were recorded. Placental and liver samples were snap-frozen in liquid nitrogen and stored at −80 °C. DNA was extracted from the fetal liver for PCR-sexing, as described in our previous study.^21^ This study used male fetuses, as they exhibit metabolic disease more clearly than female rodents.^24^ The PFOS exposure experiment was conducted three times using animals from different lots.

### Biochemical Measurement of Fasting Serum Glucose, Fatty Acids, Hepatic Triglycerides, and ATP Levels

Pregnant mice were fasted for 16 h. The fasting blood glucose levels were measured using an Accu-Check glucometer (Roche, US). A Free Fatty Acid Assay Kit (Abcam) and a Triglyceride Colorimetric Assay Kit (Cayman Chemical) were used to measure serum fatty acids and liver triglycerides, respectively. For ATP measurement, liver samples were homogenized in 1× passive lysis buffer (Promega), followed by the measurement according to the manufacturer’s instructions (ATP Determination Kit, Invitrogen). Sample protein concentrations were measured using a DC Protein Assay Kit II (Bio-Rad). Luminescence was determined by using a PerkinElmer EnSight Multimode Plate Reader.

### Measurement of Placental Cytokines

Placentas were collected and homogenized in cell lysis buffer according to the instructions of the Human Cytokine Antibody Array Kit (Abcam). Cell debris was removed from the lysate by centrifugation at 10,000 rpm for 10 min at 4 °C. The total protein concentration of the lysate was measured and diluted in a blocking buffer before membrane incubation at 4 °C overnight. After washing, the membranes were incubated with biotin-conjugated anticytokine and HRP-conjugated streptavidin solutions. Images of the chemiluminescent signals were taken. The analysis of the data was carried out using ImageJ. A triplicate antibody array experiment was conducted.

### Air-Flow Assisted Desorption Electrospray Ionization-Mass Spectrophotometry Imaging (AFADESI-MSI)

Fetuses were isolated from amniotic sacs, snap-frozen in liquid nitrogen, and stored at −80 °C. Using a Cryostar NX70 (Thermo Scientific, U.S.), the whole fetus was mounted onto a cryostat specimen chunk using 0.9% sodium chloride block, and the fetus was sliced at a thickness of 10 μm. A frozen section was mounted on a microscopic slide (Citotest, Jiangsu, China) and dried under vacuum for 15 min. The AFAI-MSI image platform (Viktor, Beijing, China) and an Orbitrap Exploris 120 mass spectrometer (Thermo Fisher, Bremen, Germany) were used to analyze the images as described in our previous studies.^25,26^ The MS data were then processed by a Thermo Xcalibur 4.5.455.18 (Thermo Fisher Scientific, U.S.). MSConvert (Nature Biotechnology Commentary) and imzMLConverter. For data visualization, SCiLSTM Lab (Bremen, Germany) was used. Afterward, hematoxylin staining was performed on the sections fixed in 4% PFA.

### Oil-Red O Staining of Fetal Liver

The cryostat NX70 was used to section liver tissues at 10 μm thickness using Cryomatrix embedding resin (Thermo Scientific). After air-drying at room temperature, the sections were stained with oil-red O and hematoxylin (Sigma-Aldrich). Quantitative analysis was conducted on four slides from the control and PFOS groups. Five randomly selected microscopic fields were quantified using ImageJ for each section.

### Whole-Genome Bisulfite Sequencing (WGBS) of Fetal Liver

Fetal hepatic DNA was isolated and fragmented by sonication with a Bioruptor (Diagenode, Belgium) to about 250 bp, followed by blunt-ending, dA addition to 3′-end, and adaptor ligation according to the manufacturer’s instructions. The EZ DNA Methylation-Gold Kit (ZYMO) was used for bisulfite conversion of ligated DNA. On a 2% TAE agarose gel, different insert-size fragments were excised. A QIAquick gel extraction kit (Qiagen) was used to purify the products, and PCR was used to amplify them. Lastly, Illumina HiSeq 4000 platforms were used for sequencing. Raw reads were filtered, including adaptor sequences, contamination, and low-quality reads. By using BSMAP, we mapped the clean reads to the mouse reference genome. The level of methylation was then calculated by dividing the total number of reads covering each methyl-cytosine (mC) by the number of reads covering that cytosine,^27^ which was also equal to the mC/C ratio at each reference cytosine.^28^ By comparing methylomes between control and treatment groups and identifying those with at least 5 CpG (CHG or CHH) sites with at least a 2-fold change, putative DMRs were determined. By comparing methylation levels of DMR by Circos,^29^ the degree of difference in methyl-cytosine (mCG, mCHG, and mCHH) between the control and treatment groups was established. From the three experimental groups, we obtained 3.99 billion clean reads, resulting in 399 Gb of sequencing data. In DNA sequences, cytosine is classified into CG, CHG, and CHH (H = A, G, or T). The sequencing depth covered all cytosine (Figure S1). We calculated the average methylation level of the whole genome based on the ratio of reads supporting methylation to reads covering a specific cytosine site (Table S1). Different genomic regions showed a greater percentage of CG methylation than CHG or CHH methylation (Table S2). The CG methylation covered over 90% of the genome’s total cytosine methylation (Figure S2). Each sample from different treatments was then examined for the methylation pattern in different gene regions. Our data showed that the methylation level of CG was in general higher than that of CHG and CHH (Figure S3). To identify dysregulation of DNA methylation, a sliding window approach was used to search for differentially methylated regions (DMRs) containing five or more CG sites. A Fisher’s exact test was used for calling methylation-enriched regions, which took into account the rate and depth of CpG methylation to construct contingency tables and determined the *p*-value.

### Real-Time PCR Analysis

Trizol reagent (Invitrogen) was used to isolate the total RNA from tissue samples. A BioDrop spectrophotometer was used to determine RNA quality and quantity. The SuperScript VILO cDNA Synthesis Kit (Applied Biosystems, Foster City, CA) was used to synthesize complementary DNA. With the StepOne real-time PCR system and Fast-SYBR Green Master Mix (Applied Biosystems), gene expression levels were determined using gene-specific primers (Table S3). The program consisted of 20 s at 95 °C, followed by 40 cycles of 95 °C for 3 s, 60 °C for 10 s, and 72 °C for 30 s. The 2^–ΔΔCt^ method was used to normalize the relative expression level with the actin transcript level. The specificity of the amplicon was verified by using melting curve analysis and agarose gel electrophoresis.

### Western Blot Analysis

Homogenized liver tissues were incubated in RIPA buffer (50 mM Tris-HCl, pH 7.4, 150 mM NaCl, 2 mM EDTA, 0.1% SDS, and 1% NP-40) supplemented with PhosStop (Sigma-Aldrich), aprotinin, and leupeptin (Sigma-Aldrich) at 2 mg/mL each. With a Bioruptor sonicator (Diagenode), the tissue homogenate was sonicated (8 s for 5 cycles) on ice. To remove cell debris, lysates were centrifuged at 11,000*g* for 15 min at 4 °C. The DC Protein Assay Kit II (Bio-Rad) was used to measure protein concentration in the supernatant using a microplate reader (BioTek). Sample lysates were resolved by SDS-PAGE and transferred to a PVDF membrane (Bio-Rad). After blocking with 5% nonfat milk in PBST for an hour, the membrane was incubated with a primary antibody, followed by an HRP-conjugated secondary antibody (Table S4). WESTSAVE Up (AbFrontier) was used to visualize specific bands.

### Human Normal Hepatocyte Cell-Line

MIHA cells were grown in Dulbecco’s modified Eagle medium (DMEM), supplemented with 10% heat-inactivated fetal bovine serum (FBS, Gibco, Life Technologies) and antibiotics (25 U/mL penicillin and 25 μg/mL streptomycin) (Life Technologies), maintained at 37 °C in a humidified, 5% CO_2_ incubator. For experiments, the cells were treated with 1, 10, and 100 μM PFOS for 24 h. PFOS levels of 100 μM (50 μg/mL) are comparable to those reached by highly PFAS-exposed individuals with PFOS levels of 92,303 ng/mL.^30^ As part of the analysis, cells were incubated with the passive lysis buffer (Promega), centrifuged for 15 min at 13,000*g* at 4 °C, and then ATP levels were measured. The cell lysates were also used for Western blot analysis.

In some experiments, cell fractionation was implemented. MIHA cells were lysed and fractionated using the Subcellular Protein Fractionation Kit (Thermo Scientific) according to the manufacturer’s instructions. Briefly, the cells were lysed with a cytoplasmic extraction buffer to extract the cytosolic fraction. This was followed by membrane extraction, and the pellet was then resuspended in a nuclear extraction buffer to extract the nuclear fraction.

### Statistical Analysis

A statistical mean and standard deviation were used to present the data. The GraphPad Prism version 8.0 was used for statistical analyses. Students’ tests were used to evaluate the physiological and gene expression data. A *p*-value <0.05 was considered statistically significant.

## Results and Discussion

### PFOS-Elicited Dysregulation of Maternal Metabolism

**Figure 1:**
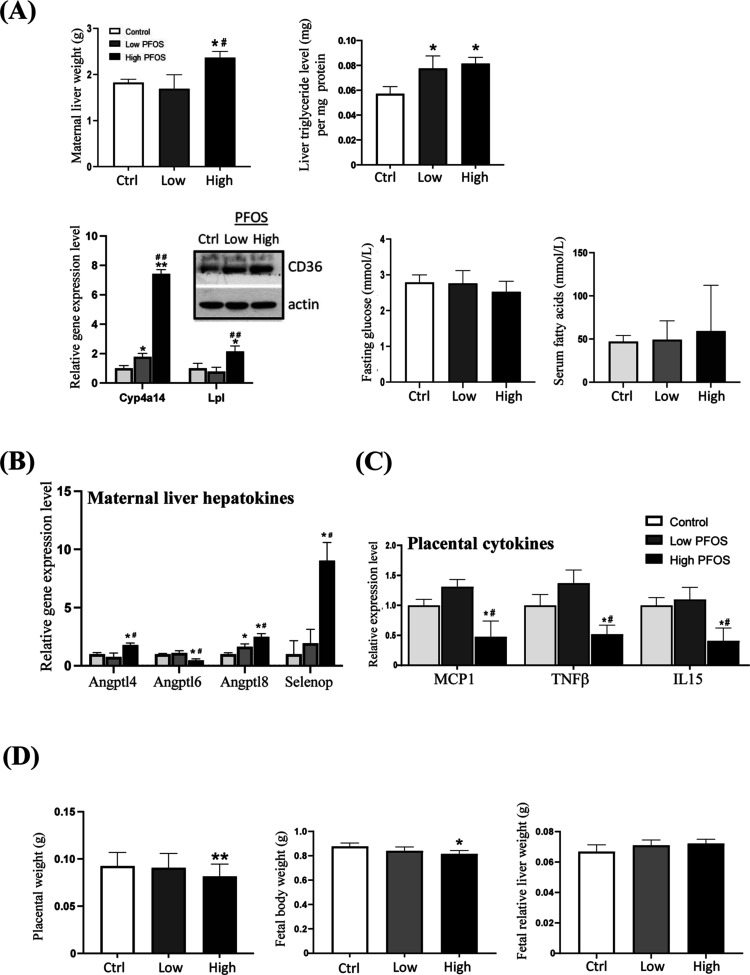
Effect of *in utero* PFOS exposure on maternal metabolism, placental and fetal body weights, and placental cytokine profiles at gestational day 17.5. (A) *Metabolic measurement of maternal mice*: the exposure caused an increase of maternal liver weights and triglyceride levels (upper panel) and an increased expression of *Cyp4a14*, *Lpl*, and Cd36 (lower panel). There was no significant change in maternal fasting serum glucose and fatty acid levels among the control and PFOS-exposed groups. (B) *Maternal hepatokines*: PFOS elicited a significant upregulation of *Angptl4*, *Angptl8*, and *Selenop* and a downregulation of *Angptl6*. (C) *Placental cytokine profiles*: a significant reduction in the expression of MCP-1, TNF-β, and IL-15 levels at the high-dose of PFOS exposure was noted. (D) *Placental and fetal body weights*: a significant reduction in placental and fetal body weights was noted at the high-dose of PFOS exposure. There was no noticeable change in the fetal relative liver weights. Data were presented as the mean ± SD *P (treatment *vs* control), ^#^*P* (low-dose *vs* high-dose) < 0.05; **P and ^##^*P* denote <0.01.

At GD 17.5, PFOS exposure caused liver hypertrophy and fat accumulation in maternal mice, as demonstrated by significant increases in liver weights and triglyceride levels (Figure 1A, upper panel). It was accompanied by significant increases of *Lpl* (lipoprotein lipase), CD36 (fatty acid transporter), and *Cyp4a14* (a gene indicative of PPARα activation) (Figure 1A, lower left panel), known to be related to ROS production.^31^ Fasting serum glucose and fatty acids did not differ significantly between the groups (Figure 1A, bottom right panel). As the liver regulates systemic energy homeostasis and the maternal and intrauterine environments are intimately connected, we examined maternal levels of liver-to-tissue messenger proteins (hepatokines), which are known to respond to perturbed glucose and lipid metabolism.^32^ Our data showed that the maternal hepatokines, angiopoietin-like (*Angptl*)-4, *Angptl-8*, and *SelenoP* were significantly upregulated while *ANgptl-6* was reduced (Figure 1B). Those hepatokines play crucial roles in lipid metabolism, inflammation, and insulin sensitivity and are essential for the development of the placenta and fetal growth.^33,34^ Although the information on the relationship of ANGPTLs and placental inflammation is limited, the emerging roles of ANGPTLs in triggering inflammatory signals in multiple tissues have been reported.^35,36^ In fact, inflammation is known to regulate placental angiogenesis, which is positively associated with the expression of cytokines.^37^ While the reduction in the expression of cytokine release from the placenta affects fetal development.^38^ Thus, we measured inflammatory signals in placentas. The cytokine array analysis showed a significant downregulation of placental monocyte chemotactic protein-1 (MCP-1), TNF-β, and IL-15 at the high dose of PFOS exposure (Figure 1C). Retrospectively, we noted that there was a significant reduction in placental and fetal body weights at the high dose (3 μg/g) of PFOS exposure (Figure 1D). There was no significant change in the relative fetal liver weights. The data showed that *in utero* exposure to PFOS reduced placental and fetal weights, suggesting disruptions in the metabolic programming of the fetuses. The next step was to examine possible changes in the epigenetic landscape of fetal livers.

### Whole-Genome Bisulfite Sequencing (WGBS) of Fetal Livers at Gestation Day 17.5

**Figure 2:**
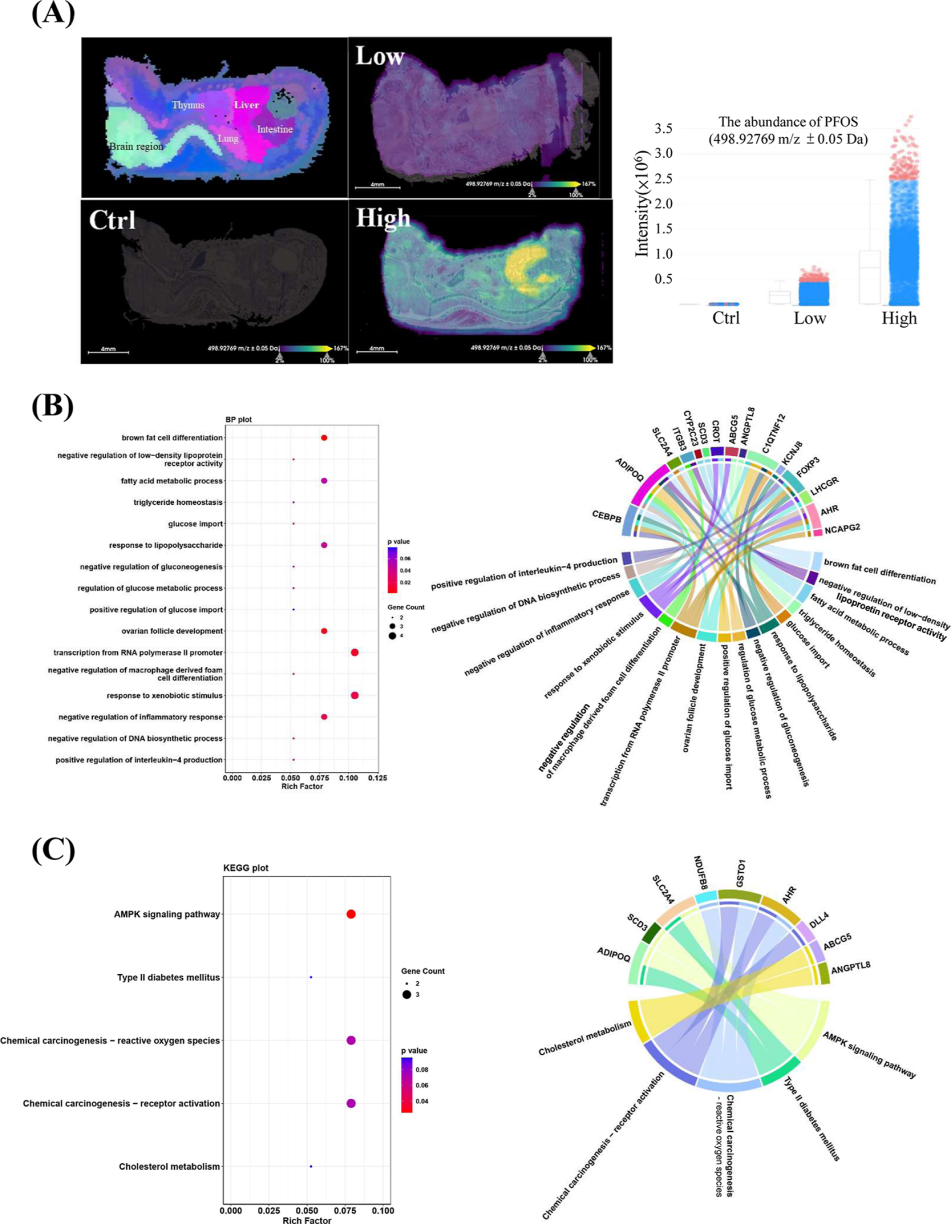
AFADESI-mass spectrophotometry imaging of PFOS-distribution of fetuses and whole-genome bisulfite sequencing (WGBS) of fetal livers at gestational day 17.5. (A) *AFADESI-MS imaging*: left-top corner, the annotation of the major tissues of the control fetus. Ion image of PFOS distribution in different fetal regions in control and PFOS-exposed groups (left-bottom and right-top and bottom). A box and dot plot showed the signal intensity of PFOS (498.92769 *m*/*z* ± 0.05 Da) in the cross-section of whole fetuses. The box represents the lower and upper quantiles. Signal intensities are represented by blue dots, while outliers are represented by red dots. (B) *WGBS-gene ontology (GO): the biological functions and signaling pathways of fetal hepatic genes, commonly identified in both low- and high-dose PFOS-exposed groups*. The left panel: an enrichment analysis highlighted the involvement of PFOS-elicited DMR genes in biological processes related to fatty acid and glucose metabolism and inflammatory responses. The rich factor for each functional term (*y*-axis) was calculated as the number of DMR genes annotated to the terms divided by the number of reference genes annotated to those terms. The size of the bubble represented the number of DMR genes. The color of the bubble represented the significance of the processes. The right panel: the Circos plot showed the relationships and interactions of DMR genes in the highlighted biological processes. Along the circle’s circumference, different data tracks are displayed in a circular layout. A track displays the gene name, and another displays functional categories or pathways. (C) *WGBS-KEGG analysis*: the left panel: an enrichment analysis highlighted the involvement of PFOS-elicited DMR genes in cholesterol metabolism, chemical carcinogenesis, type II diabetes mellitus, and the AMPK pathway. The right panel: the Circos plot showed the involvement of DMRs in the highlighted signaling pathways.

Whole-fetus mass spectrophotometry imaging revealed that PFOS was selectively accumulated in fetal livers (Figure 2A), indicating that possible chemical perturbation occurred in the livers *in situ*. Fetal livers were then subjected to WGBS, which revealed that *in utero* PFOS exposure perturbed epigenetic modifications of genes associated with inflammation and energy metabolism (Figure 2B). In comparing the control and low-dose PFOS groups, we identified 168 DMRs, including 93 hyper-methylated and 75 hypo-methylated regions at the proximal promoters (2kb) of protein-coding genes (Table S5). The control and high-dose PFOS comparison resulted in 272 DMRs, including 114 hyper-methylated and 158 hypo-methylated regions of protein-coding genes (Table S6). Based on the overlap of the DMRs from the low- and high-dose PFOS groups, 26 candidate genes were identified as commonly hyper-methylated and 13 genes as commonly hypo-methylated in the fetal livers (Supporting Information Table 1). Gene ontology analysis showed that PFOS treatment altered genes related to the fatty acid metabolism (*CEBPB, ADIPOQ, SLC2A4, ITGB3, CYP2C23, SCD3, CROT, ABCG5, and ANGPTL8*) and glucose metabolism (*C1QTNF12, SLC2A4, CEBPB, KCNJ8, FOXP3*, and *ADIPOQ*), in affecting biological processes such as the differentiation of brown fat cells, the homeostatic regulation of triglycerides, glucose import, gluconeogenesis, and inflammatory responses (Figure 2B). KEGG enrichment analysis identified the roles of *ADIPOQ, SCD3*, and *SLC2A4* in type-II diabetes, cholesterol metabolism, and the AMPK signaling pathway (Figure 2C). Analyzing hypo-methylated and hyper-methylated genes separately identified similar enriched biological functions and processes (Figure S4).

**Figure 3:**
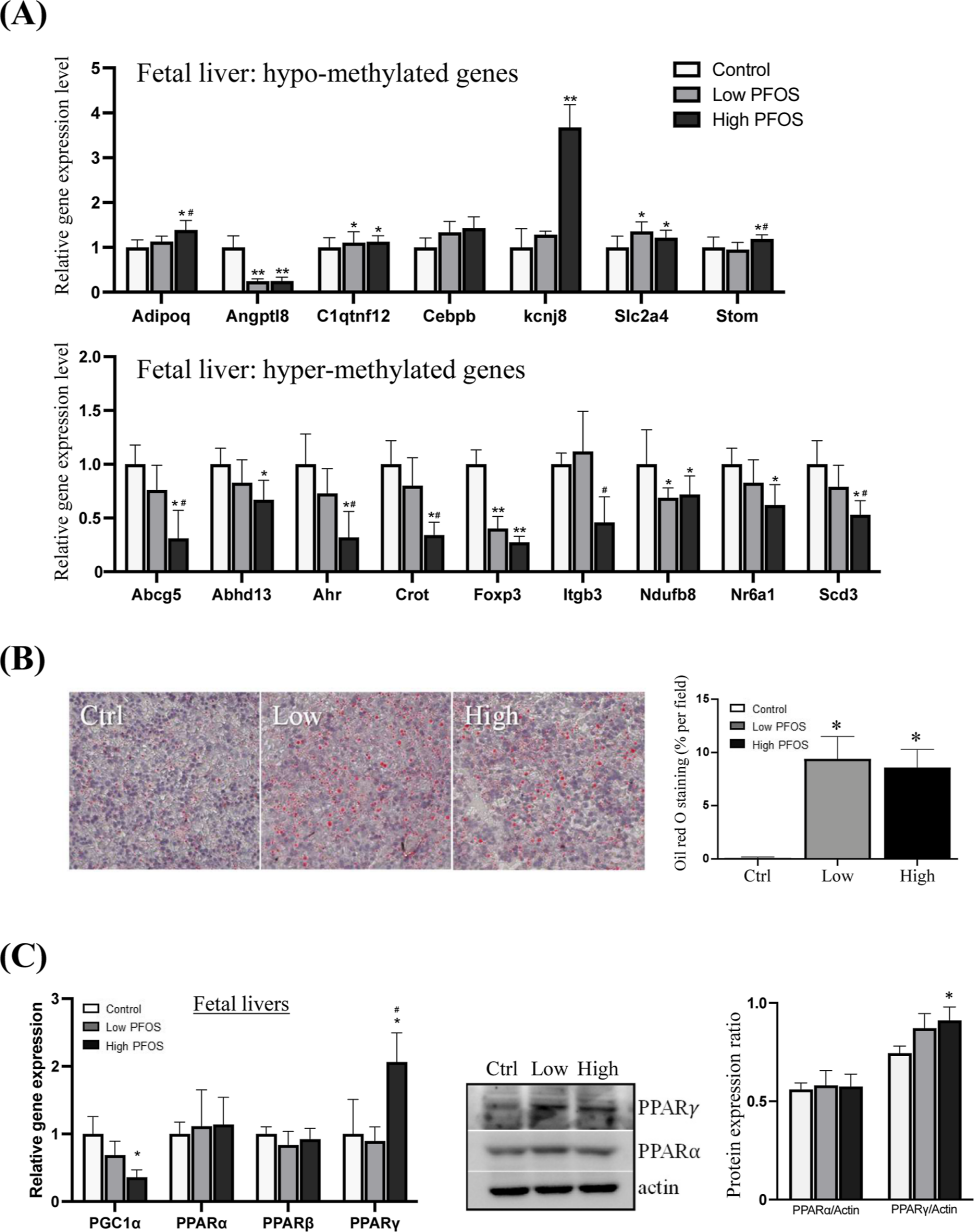
Differential expression of hypo-, hyper-methylated, and PPAR genes in fetal livers at gestational day 17.5. (A) *Validation of WGBS data*: analysis of differentially methylated gene clusters in fetal livers commonly identified in both low- and high-dose *in utero* PFOS exposure using real-time PCR. The upper panel: there were significant elevated expression levels of the hypo-methylated genes. The lower panel, the expression levels of hyper-methylated genes were significantly reduced. (B) Significant increases in the number of hepatic microvesicular lipid droplets were noted in PFOS-exposed fetuses. (C) *Expression profiles of PPARs and PGC1*α *in fetal livers*. Left panel: A significant increase but a decrease in the expression levels of *Ppar*γ and *Pgc1*α, respectively, were noted in high-dose PFOS-exposed groups. Right panel: Western blot showed a significant increase of PPARγ. Data were presented as the mean ± SD *P (treatment *vs* control), ^#^*P* (low-dose *vs* high-dose) < 0.05; **P and ^##^*P* denote <0.01.

To validate WGBS data for gene expression, transcript levels of the 16 commonly DMR genes were chosen for further analysis. Consistently, significant increased expression of the hypo-methylated and decreased expression of the hyper-methylated genes were noted (Figure 3A). The higher expression levels of *Cebpb* [CCAAT/enhancer-binding protein (C/EBP), a pivotal transcriptional factor to increase hepatic lipid metabolism],^39^*Adipoq* (adiponectin) and *C1atnf12* (C1q and tumor necrosis factor related 12, hepatic genes critical for glucose and lipid metabolism),^40,41^*Stom* (stomatin, a major constituent of lipid raft, to promote fatty acid uptake),^42^*Slc2a4* [solute carrier family 2 (member 4)—Glut4], and the decreased expression of *Abhd13* (α/β-hydrolase fold domain protein, lipid-metabolizing enzymes),^43^*Scd3* (stearoyl-coenzyme A desaturase-3, lipid synthesis at early liver development),^44^*Ndufb8* (NADH:ubiquinone oxidoreductase subunit B8, mitochondrial oxidative-phosphorylation),^45^*Crot* (carnitine-*O*-octanoyl-transferase, a peroxisomal enzyme for oxidation of very long-chain fatty acids),^46^*Abcg5* (ATP-binding cassette subfamily G member 5, in cholesterol excretion)^47^ were associated with the perturbation of glucose and lipid metabolism. Additionally, the reduced expression of *Ahr* (aryl hydrocarbon receptor, in xenobiotic response and hepatic steatosis)^48^ and *Nr6a1* (nuclear receptor subfamily 6, a regulator of lipid metabolism)^49^ increased the chance of hepatic lipid accumulation. However, it was observed that for the hypo-methylated gene, *Angptl8*, its expression level was significantly reduced in the livers of PFOS-exposed fetuses. Given the circumstances of decreased placenta nutrient transfer^20^ and the reduced placental and fetal body weights (Figure 1D), the decreased *Angptl8* expression would be possibly regulated negatively by nutrition availability.^50^ Apparently, the reduced fetal hepatic *Angptl8* expression levels were reported to alleviate insulin resistance in the placenta,^51^ leading to perturbations of fetal metabolic health. Herein, the predicted biological outcomes were manifested with an increase of fetal hepatic lipid accumulation (Figure 3B), which was also supported by the data of a significant increase in the expression of the lipogenic transcriptional factor, PPARγ and a significant reduction in the expression of PPAR-γ coactivator 1α (*Pgc1*α) (Figure 3C), a transcriptional factor to reduce triacylglycerol storage.^52^ Although PPARγ was mostly expressed in adipocytes, a previous study suggested that the liver expressed considerable amounts of PPARγ in the presence of hepatic lipid accumulation,^53^ resulting in maladaptive changes to metabolic function. Moreover, there was possibility that PFOS modulated both PPARα and PPARγ activities,^54^ that simultaneously stimulated the activity of β-oxidation and lipogenesis. This deranged stimulation was reported in numerous studies on fatty liver disease.^55,56^ A follow-up study then examined downstream targets for inflammation and metabolism in fetal livers.

### *In Utero* PFOS Exposure Dysregulated Energy Sensing and Prompted Lipogenesis in Fetal Livers

**Figure 4:**
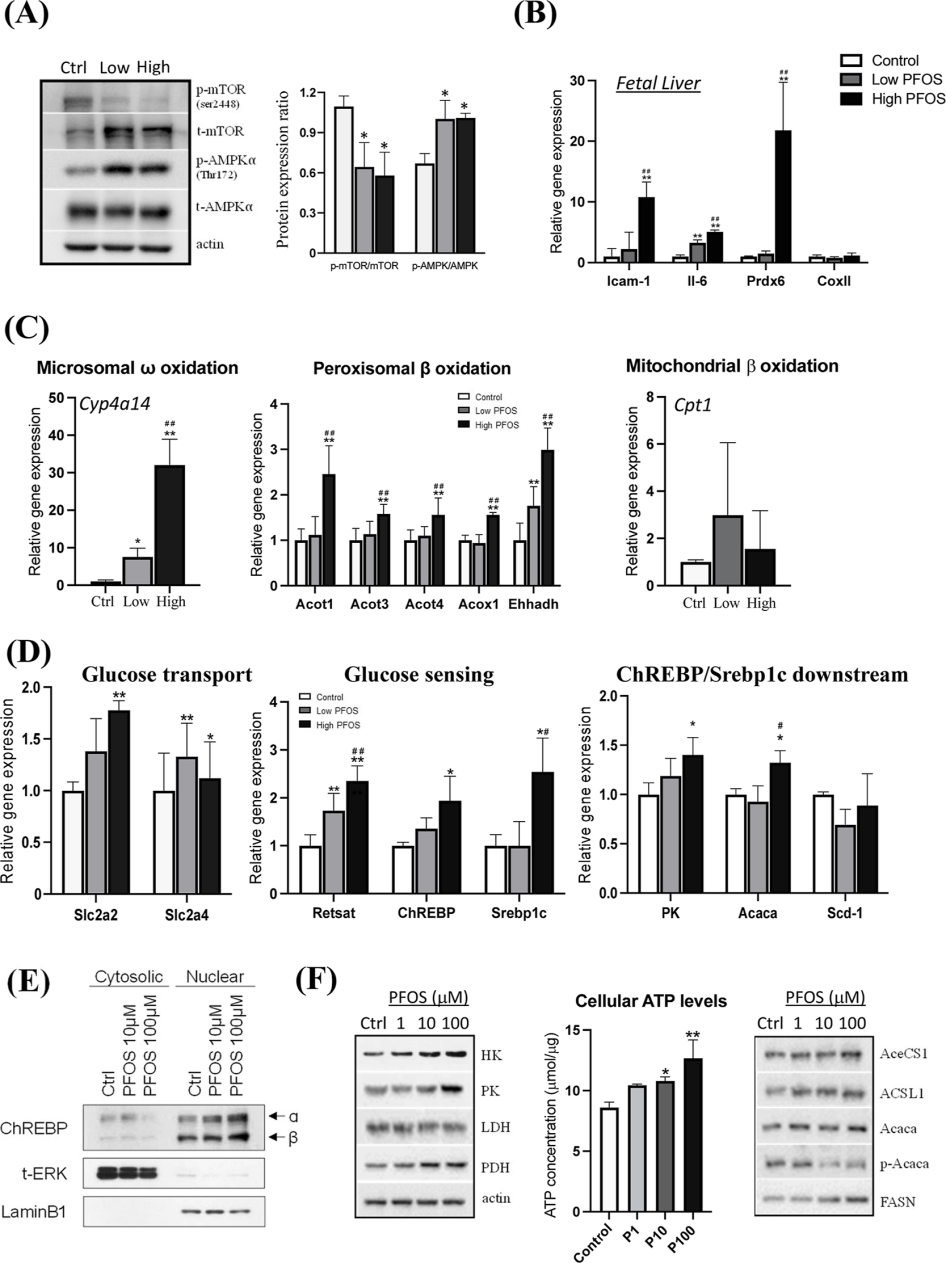
*In utero* PFOS exposure perturbed energy sensing, inflammation, and glucose-fatty acid metabolic gene expression in fetal livers at gestational day 17.5 and in the human normal liver cell-line. (A) PFOS exposure significantly increased and decreased pAMPK/AMPK and p-mTOR/mTOR levels, respectively, in the livers of fetuses. (B) The master markers of inflammation *Icam-1*, interleukin 6 (*Il6*), and the antioxidant enzyme peroxiredoxin 6 (*Prdx6*) were significantly increased in PFOS-exposed groups. (C) Hepatic mRNA expression of key genes in microsomal ω- and peroxisomal β-oxidation was significant upregulation in PFOS-exposed groups. (D) The expression of rate-limiting metabolic genes involved in glucose transport [*Slc2a2* (*Glut2*), *Slc2a4* (*Glut4*)], glucose-sensing (*Retsat, ChREBP*), glycolytic enzyme (pyruvate kinase, *Pk*), and lipogenesis (*Srebp1c, Acaca*) in the fetal livers was significantly upregulated in PFOS-exposed groups. (E) *Human normal hepatocyte cells (MIHA)*: PFOS treatment increased the nuclear translocation of ChREBP, a glucose-sensing transcription factor. As the loading controls, cytosolic and nuclear markers are total ERK and laminB1, respectively. (F) *Human normal hepatocyte cells (MIHA)*: a significant increase in ATP levels was associated with upregulation of glycolytic and fatty acid synthesis enzymes in PFOS treatment. Data were presented as the mean ± SD *P (treatment *vs* control), ^#^*P* (low-dose *vs* high-dose) < 0.05; **P and ^##^*P* denote <0.01.

As a result of fetal lipid accumulation, PFOS-exposed fetuses showed significant increases in hepatic ATP levels (Figure S5A). On the contrary, maternal hepatic ATP levels were significantly lower in PFOS-exposed groups (Figure S5B). The reduction in maternal hepatic ATP levels might be due to PFOS-induced mitochondrial dysfunction, inflammation, and insulin resistance, as reported in previous studies.^8,57^ Comparatively, fetal livers exhibited mild pathological symptoms. It is imperative to note that the severity of the hepatic lipid accumulation and its ability to adapt to changing metabolic states determine the specific changes in liver ATP levels.^58^ In this study, the increase of fetal hepatic ATP levels might be associated with PFOS-elicited increase of PPAR signaling pathways, in particularly of PPARγ, which was suggested to increase ATP production.^59^ Western blot analysis of liver samples of fetuses exposed to PFOS revealed that mTOR/AMPK signaling was significantly altered (Figure 4A). The inhibition of fetal hepatic mTOR-signaling was associated with decreases in protein synthesis and cell growth,^60^ which is in line with our data, showing the placenta and fetal body weights were significantly decreased. The activation of AMPK may be a way to compensate for the metabolic disruption caused by PFOS to increase ATP production through ROS-induced cellular responses, glucose, and fatty acid metabolism.^61–63^ A stimulation of fetal hepatic oxidative stress was illustrated by an upregulation of the master regulator of cellular responses in inflammation *Icam-1*, the inflammatory marker interleukin 6 (*Il6*), and the antioxidant enzyme against oxidative stress and inflammation peroxiredoxin 6 (*Prdx6*) (Figure 4B). Moreover, the manifestation of oxidative stress^64,65^ was exemplified with the upregulation of the PPARα-target genes, [microsomal ω-oxidation (*i.e.*, *Cyp4a14*) and peroxisomal β-oxidation (*i.e.*, *Acot1, Acot3, Ehhadh*)] (Figure 4C). However, there were no significant changes in the expression level of the rate-limiting enzyme, *Cpt1* for mitochondrial β-oxidation (Figure 4C, right panel). Nevertheless, the dysregulation of mTOR/AMPK and of PPARγ pathways was linked with the progression of NAFLD.^66^

AMPK-activation promoted glycolysis, while PPARγ regulated *Retsat* (retinol saturase), which is the upstream regulator of the transcriptional factor, *ChREBP* (the carbohydrate-responsive element binding protein), that promotes glycolysis and lipogenesis.^67,68^ Our data showed significant increases in the expression levels of the glucose transporters (*Glut2 and Glut4*) (Figure 4D, left panel), which might support glucose utilization in PFOS-induced metabolic disturbances. The increase of glucose uptake was accompanied by significant upregulation of the glucose sensors (*Retsat* and *ChREBP*) and the transcriptional factor for lipid synthesis [sterol regulatory element-binding protein, *Srebp1c*] (Figure 4D, middle panel).^69^ It was reported that *ChREBP* coordinated with *Srebp1c* for liver lipogenesis.^70^ Consistently, our data showed that the *ChREBP/Srebp1c*-downstream targets, pyruvate kinase (*Pk*) and acetylCoA carboxylase (*Acaca*) were significantly upregulated (Figure 4D, right panel). We then characterized the underlying mechanism for PFOS-induced fatty acid synthesis using the human normal hepatocyte cell line (MIHA) to illustrate metabolic changes, including glycolysis and fatty acid synthesis.

PFOS treatment on MIHA cells activated the nuclear translocation of the transcriptional factor, ChREBP (Figures 4E and S6A), which is the positive regulator of the glycolytic enzymes, PK and the lipogenic enzymes, ACACA and fatty acid synthase (FASN).^67,68^ According to the Western blot analysis, PFOS treatment significantly increased the expression levels of the first and terminal glycolytic enzymes (hexokinase, HK, and PK) and the glycolysis-Krebs cycle’s link-reaction enzyme, pyruvate dehydrogenase (PDH) (Figure 4F, left panel and Figure S6B). Moreover, a significant reduction in the expression level of lactate dehydrogenase (LDHA) was noted. Modulation of glycolysis and Krebs cycle enzymes was reported to affect NAFLD development.^71,72^ Apparently the data implied an increase of glucose catabolism in mitochondria, which was aligned with a dose-dependent increase in cellular ATP levels (Figure 4F, middle panel). For fatty acid synthesis, both cytoplasmic acetyl-CoA synthetase (AceCS1) and mitochondrial acyl-CoA synthetase long-chain family member 1 (ACSL1) were significantly upregulated (Figure 4F, right panel and Figure S6B). These enzymes are known to convert acetate into acetylCoA, which serves as the building block and substrate of ACACA and FASN,^73^ that were also significantly upregulated (Figure 4F). Collectively, the animal and human cell-line data supported the effects of PFOS on the upregulation of glucose catabolism and fatty acid synthesis.

In summary, *in utero* PFOS exposure disrupted maternal liver metabolism, affecting hepatokine release and reducing placental and fetal growth. WGBS revealed dysregulation of the DNA methylation of genes related to inflammation, glucose metabolism, and fatty acid metabolism in fetal livers. There was a correlation between fetal metabolism perturbation and the stimulation of PPAR-α and -γ downstream targets and the activation of AMPK signaling. This resulted in the dysregulation of glucose and lipid metabolism *via* the simultaneous stimulation of glucose catabolism, fatty acid oxidation, and fatty acid synthesis. The deranged stimulation of glucose uptake and the retsat-ChREBP/Srebp1c-Pk-Acaca-Fasn pathway caused lipid accumulation in fetal livers. The altered maternal-placental-fetus feedback circuitry led to maladaptation of fetal metabolism, adversely affecting fetal liver development and metabolic health.

## Supporting Information Available

Commonly hyper- and hypo-methylated genes in fetal livers exposed to low- and high-dose PFOS in-utero (XLSX)
Average methylation level of the whole genome, covering specific cytosine sites; average methylation level in different genomic regions, covering specific cytosine sites; list of real-time PCR primers; list of antibodies; methylation profiles of the control group and low-dose PFOS group, at the proximal promoter (2kb) of protein-coding genes; methylation profiles of the control group and high-dose PFOS group, at the proximal promoter (2kb) of protein-coding genes; cumulative coverage of the corresponding depth in WGBS of livers of fetuses exposed to low- and high-dose PFOS *in utero*; proportion of different types of methylated cytosine in WGBS of livers of fetuses exposed to low- and high-dose PFOS *in utero*; methylation trend in gene regions in WGBS of livers of fetuses exposed to low- and high-dose PFOS *in utero*; WGBS-gene ontology: the biological functions and signaling pathways of fetal hepatic genes, commonly identified in both low- and high-dose PFOS-exposed groups; fetal and maternal hepatic ATP levels at gestational day 17.5; and statistical analysis of Western blot data from MIHA cells treated with PFOS (PDF)
