# Supplementary material for: Effects of In Utero PFOS Exposure on Epigenetics and Metabolism in Mouse Fetal Livers
Source: Environ Sci Technol. 2023 Sep 27;57(40):14892–903. doi: 10.1021/acs.est.3c05207 (PMC10569047; doi:10.1021/acs.est.3c05207)
Supplement: Supplementary file 2 — es3c05207_si_002.pdf [file es3c05207_si_002.pdf]

Effects of *in-utero* PFOS exposure on epigenetics and metabolism in mouse fetal livers

HO Tsz Chun[1, 2], WAN Hin Ting[1], LEE Wang Ka[1], LAM Thomas Ka Yam[2], LIN Xiao[3], CHAN Ting Fung[4], LAI Keng Po[5], WONG Chris Kong Chu[1,2*]

[1]Croucher Institute for Environmental Sciences, Department of Biology, Hong Kong Baptist University, Hong Kong SAR;

[2]State Key Laboratory in Environmental and Biological Analysis, Hong Kong Baptist University, Hong Kong SAR;

[3]Department of Psychiatry, Icahn School of Medicine at Mount Sinai, New York, USA

[4]School of Life Sciences, State Key Laboratory of Agrobiotechnology, Bioinformatics Centre, the Chinese University of Hong Kong, Hong Kong SAR;

[5]Key Laboratory of Environmental Pollution and Integrative Omics, Guilin Medical University, Education Department of Guangxi Zhuang Autonomous Region, Guilin, China.

Supplementary Files

Supplementary Table S1. The average methylation level of the whole genome, covering specific cytosine sites**.**

Supplementary Table S2. The average methylation level in different genomic regions, covering specific cytosine sites.

Supplementary Table S3. A list of real-time PCR primers.

Supplementary Table S4. A list of antibodies.

Supplementary Table S5. The methylation profiles of the control group and low-dose PFOS group, at the proximal promoter (2kb) of protein-coding genes.

Supplementary Table S6. The methylation profiles of the control group and high-dose PFOS group, at the proximal promoter (2kb) of protein-coding genes.

Supplementary Fig S1. Cumulative coverage of corresponding depth in WGBS of livers of fetuses exposed to low (0.3 μg/g bw) and high-dose (3 μg/g bw) PFOS *in-utero*.

Supplementary Fig S2. The proportion of different types of methylated cytosine in WGBS of livers of fetuses exposed to low (0.3 μg/g bw) and high-dose (3 μg/g bw) PFOS *in-utero*.

Supplementary Fig S3. Methylation trend in gene regions in WGBS of livers of fetuses exposed to low (0.3 μg/g bw) and high-dose (3 μg/g bw) PFOS *in-utero*.

Supplementary Fig S4. WGBS-Gene Ontology (GO): the biological functions and signaling pathways of fetal hepatic genes, commonly identified in both low- and high-dose PFOS-exposed groups.

Supplementary Fig S5. Fetal and Maternal hepatic ATP levels at gestational day 17.5.

Supplementary Fig S6. A statistical analysis of western blot data from MIHA cells treated with PFOS.

Supplementary Table S1. The average methylation levels of the whole genome, covering specific cytosine sites.

Chr	C (%)	CG (%)	CHG (%)	CHH (%)
chr1	3.8780	71.1227	1.0330	1.0337
chr10	4.3100	72.6710	0.9973	1.0033
chr11	4.2410	68.3533	1.0350	1.0380
chr12	3.9967	71.0380	1.0197	1.0297
chr13	4.1020	71.9587	1.0107	1.0203
chr14	4.1193	71.8750	1.0260	1.0840
chr15	4.1473	71.3060	0.9980	1.0027
chr16	4.0200	71.9977	0.9813	0.9927
chr17	4.3027	66.9887	1.0160	1.0177
chr18	4.0810	72.5040	0.9933	1.0040
chr19	4.3697	69.9870	1.0203	1.0307
chr2	4.2420	70.6797	1.0277	1.0393
chr3	3.8860	71.7663	0.9863	0.9953
chr4	4.0863	70.1850	1.0117	1.0120
chr5	4.2577	70.8003	0.9990	1.0037
chr6	3.9307	71.4503	1.0167	1.0133
chr7	3.9183	68.7660	0.9947	0.9987
chr8	4.2073	70.4277	1.0187	1.0207
chr9	4.7853	72.0200	1.0970	1.2077
chrM	0.8367	0.6890	0.8437	0.8620
chrX	3.2547	71.7803	0.9607	0.9570
chrY	3.0567	61.6197	1.0867	1.0590
Whole Genome	4.1017	70.1997	1.0147	1.0277

Supplementary Table S2. The average methylation level in different genomic regions, covering specific cytosine sites.

Different Genome Regions	C (%)	CG (%)	CHG (%)	CHH (%)
5-UTR	4.03	17.46	0.95	0.96
CDS	8.68	67.65	1.06	1.05
intron	4.30	72.99	1.03	1.04
3-UTR	5.31	73.56	1.05	1.06
mRNA	4.54	71.22	1.03	1.04
ncRNA	4.20	67.52	1.02	1.03
transposons	3.90	75.58	1.03	1.05

Supplementary Table S3: A list of real-time PCR primers.

Primer	Forward	Reverse
Abcg5	CTGGAAGGGGAGGTGTTTGT	GCTGTGTATCGCAACGTCTC
Abhd13	CAGGCCCACTTTATGAAGCC	GTAACAGAGAGATGCGGCAG
Acaca	CAGAGAGTTCACCCAGCAGA	CCAGGTGTCGATAAATGCGG
Acot1	GCTATGGCCTCCTTCCTGAA	TCCACGGGAATGAAGCTCTT
Acot3	AAGAGCTTGATTCCCGTGGA	ACAGTGGGAAGTAAGGAGGC
Acot4	GGCTTTCTCTGGCATTCTGG	TAAGAGAGTACCTGGGGCCT
Acox1	GGACCTTCACTTGGGCATGTT	ATCTCCAGATTCCAGGCCGG
Actin	TCTACGAGGGCTATGCTCTCC	TCTTTGATGTCACGCACGATTTC
Adipoq	GGTCCTAAGGGTGAGACAGG	AGTCCCGGAATGTTGCAGTA
Ahr	TGCACAAGGAGTGGACGA	AGGAAGCTGGTCTGGGGTAT
Angptl8	GCTTTACACCTTCGAGCTGA	ATCCAGGTAGTCTCAGGCTG
C1qtnf12	GCGTGTGGATTCCCCCAATA	CCGTCGGACAAAGTTCAACC
Cebpb	TCTACTACGAGCCCGACTGC	GGTAGGGGCTGAAGTCGAT
ChREBP	CTGGGGACCTAAACAGGAGC	GAAGCCACCCTATAGCTCCC
CoxII	CTCCTCAATACTGGAAGCCG	ATCTAGTCTGGAGTGGGAGG
Crot	GATCCATCAGTACGCTGGGG	TCTGGCAATGGTATGTCCCG
Cyp4a14	ATTGGTTATGGTTTGCTCCTGTTG	TCATAGTGGAAGGCTCCAGTCA
Ehhadh	ATGTGGGTTGGAAAGTTCGC	ACCAGCCCTTACCTGTCTTC
Foxp3	CTCATGATAGTGCCTGTGTCCTCAA	AGGGCCAGCATAGGTGCAAG
Icam-1	CAATTTCTCATGCCGCACAG	AGCTGGAAGATCGAAAGTCCG
Il-6	ACAAAGCCAGAGTCCTTCAGA	TGGTCCTTAGCCACTCCTTC
Itgb3	GGGCGTTGTTGTTGGAGAG	ACAAAGTCTCATCTGAGCACCAG
Kcnj8	CGCAAACCCGAGTCTTCTAGGA	CCTGGCCAACATCTTCCTTTCAC
Lpl	ATCAACTGGATGGAGGAGGAGT	TTCTTATTGGTCAGACTTCCTGCT
Ndufb8	GATCCAGGTCCTTGTCCTTCC	ACAGCGGATCCTTCACTCTC
Nr6a1	AATGTCTGGGCAGTGTCTGG	TAGGACTGTGGGAGGACTGG
Pgc1α	CTGCATGAGTGTGTGCTGTG	ATCTGGGCAAAGAGGCTGG
Pk	CTTGCTCTACCGTGAGCCTC	ACCACAATCACCAGATCACC
Pparα	ACCTTGTGTATGGCCGAGAA	AAGGAGGACAGCATCGTGAA
Pparβ	AGTGACCTGGCGCTCTTCAT	CGCAGAATGGTGTCCTGGAT
Pparγ	CGCTGATGCACTGCCTATGA	AGAGGTCCACAGAGCTGATTCC
Prdx6	TTGATGATAAGGGCAGGGAC	CTACCATCACGCTCTCTCCC
Retsat	GACCGATCCACAATGACTGC	CCAGTCACACTCTCCACCTT
Scd1	CGAGAGAAGGTGAAGACGGT	GCAGCAGGACCATGAGAATG
Scd3	GCCAGCTGGATAGGGGTTTA	AGGTCCCTCGGATACAAGAC
Slc2a2	CCCAGCAGTTCTCAGGAATC	CTCACGTAACTCATCCAGGC
Slc2a4	GACGGACACTCCATCTGTTG	GCCACGATGGAGACATAGC
Srepb1c	CCCACCTCAAACCTGGATCT	AAGCAGCAAGATGTCCTCCT
Stom	GGGTGGAGATTAAGGACGTG	CCCCTTCAGCTGCAATCA

Supplementary Table S4: A list of antibodies.

Antibody	Host	Catalog, company
Acaca	Rabbit	#3676, Cell Signaling Tech.
p-Acaca	Rabbit	#11818, Cell Signaling Tech.
AceCS1	Rabbit	#3658, Cell Signaling Tech.
ACSL1	Rabbit	#9189, Cell Signaling Tech.
Actin	Mouse	A2228, Sigma
AMPKα	Rabbit	#5832, Cell Signaling Tech.
p-AMPKα (Thr172)	Rabbit	#2535, Cell Signaling Tech.
CD36	Rabbit	PA1-16813, Invitrogen
FASN	Rabbit	#3180, Cell Signaling Tech.
HK	Rabbit	#2867, Cell Signaling Tech.
Lamin B1	Rabbit	Ab16048, Abcam
LDH	Rabbit	#3582, Cell Signaling Tech.
P44/42 MAPK (Erk1/2)	Rabbit	#9102, Cell Signaling Tech.
mTOR	Rabbit	#2983, Cell Signaling Tech.
p-mTOR (S2448)	Rabbit	#5536, Cell Signaling Tech.
PDH	Rabbit	#3205, Cell Signaling Tech.
PK	Rabbit	#4053, Cell Signaling Tech.
PPARα	Rabbit	Ab24509, Abcam
PPARγ	Rabbit	Ab209350, Abcam

Supplementary Table S5. The methylation profiles of the control group and low-dose PFOS group, at the proximal promoter (2kb) of protein-coding genes.

Chr	Start	End	p-value	Ave CpG methylation (Ctrl)	Ave CpG methylation rate (low dose PFOS)	low dose PFOS/Ctrl	Gene ID	Gene name	hyper/hypo
chr8	78508029	78508870	1.03E-222	0.25	0.01	0.040	ENSMUSG00000031684.11	Slc10a7	hypo
chr7	102619578	102619804	1.24E-08	0.24	0.01	0.042	ENSMUSG00000073972.2	Olfr553	hypo
chr18	74215531	74215816	3.44E-07	0.21	0.05	0.24	ENSMUSG00000024560.7	Cxxc1	hypo
chr7	108174275	108174783	8.86E-14	0.69	0.19	0.28	ENSMUSG00000096068.1	Olfr486	hypo
chr7	103037411	103037679	1.12E-05	0.83	0.28	0.34	ENSMUSG00000073961.4	Olfr582	hypo
chr2	119324201	119324433	4.39E-06	0.28	0.1	0.36	ENSMUSG00000027314.6	Dll4	hypo
chr9	24096733	24097136	3.66E-07	0.38	0.14	0.37	ENSMUSG00000043659.11	Npsr1	hypo
chr10	77620805	77621071	7.53E-15	0.82	0.31	0.38	ENSMUSG00000009293.17	Ube2g2	hypo
chr15	75215911	75216184	5.17E-07	0.29	0.11	0.38	ENSMUSG00000047728.13	Ly6g2	hypo
chr2	158623505	158623913	2.13E-16	0.84	0.32	0.38	ENSMUSG00000037761.16	Actr5	hypo
chr1	174084271	174084773	5.45E-11	0.58	0.23	0.40	ENSMUSG00000049528.7	Olfr429	hypo
chr2	148409046	148409546	1.40E-07	0.2	0.08	0.40	ENSMUSG00000074743.4	Thbd	hypo
chr2	119324197	119324433	1.84E-05	0.27	0.11	0.41	ENSMUSG00000027314.6	Dll4	hypo
chr12	71136488	71136730	3.15E-26	0.29	0.12	0.41	ENSMUSG00000021079.16	Timm9	hypo
chr12	71136488	71136730	3.15E-26	0.29	0.12	0.41	ENSMUSG00000034601.17	2700049A03Rik	hypo
chr1	21240568	21240878	1.02E-03	0.31	0.13	0.42	ENSMUSG00000025934.15	Gsta3	hypo
chr7	102720502	102720991	4.74E-10	0.59	0.26	0.44	ENSMUSG00000066273.6	Olfr33	hypo
chrX	73064485	73064723	2.22E-04	0.36	0.16	0.44	ENSMUSG00000046287.7	Pnma3	hypo
chr2	167687888	167688109	4.44E-07	0.38	0.17	0.45	ENSMUSG00000056501.3	Cebpb	hypo
chr7	46030496	46030768	9.51E-08	0.29	0.13	0.45	ENSMUSG00000030834.7	Abcc6	hypo
chr11	69948988	69949374	2.81E-07	0.2	0.09	0.45	ENSMUSG00000018566.14	Slc2a4	hypo
chr7	102687428	102687847	4.10E-11	0.31	0.14	0.45	ENSMUSG00000073968.4	Trim68	hypo
chr15	4897768	4898211	4.80E-10	0.77	0.35	0.45	ENSMUSG00000022155.9	Mroh2b	hypo
chr18	53244275	53244489	2.89E-03	0.22	0.1	0.45	ENSMUSG00000024535.16	Snx24	hypo
chr19	4477295	4477640	3.55E-05	0.22	0.1	0.45	ENSMUSG00000049303.10	Syt12	hypo
chr4	126325470	126325697	5.70E-07	0.24	0.11	0.46	ENSMUSG00000028845.15	Tekt2	hypo
chr6	52192691	52192897	5.39E-06	0.24	0.11	0.46	ENSMUSG00000000942.10	Hoxa4	hypo
chr7	102571148	102571469	4.52E-10	0.61	0.28	0.46	ENSMUSG00000073975.3	Olfr550	hypo
chr14	19750407	19750849	2.42E-10	0.26	0.12	0.46	ENSMUSG00000021806.4	Nid2	hypo
chr1	133309280	133309828	2.14E-10	0.39	0.18	0.46	ENSMUSG00000103421.1	Golt1a	hypo
chr1	133309280	133309828	2.14E-10	0.39	0.18	0.46	ENSMUSG00000115958.1	Gm28040	hypo
chr1	133309280	133309828	2.14E-10	0.39	0.18	0.46	ENSMUSG00000098306.9	Gm28040	hypo
chr5	119668855	119669219	2.66E-11	0.26	0.12	0.46	ENSMUSG00000018604.18	Tbx3	hypo
chr16	98082652	98083082	4.85E-08	0.41	0.19	0.46	ENSMUSG00000116673.1	A630089N07Rik	hypo
chr11	117827081	117827540	5.63E-06	0.28	0.13	0.46	ENSMUSG00000025574.13	Tk1	hypo
chr5	119668654	119669219	3.27E-17	0.28	0.13	0.46	ENSMUSG00000018604.18	Tbx3	hypo
chrX	157818047	157818264	1.56E-04	0.28	0.13	0.46	ENSMUSG00000047485.6	Klhl34	hypo
chr2	153779559	153779905	2.80E-07	0.47	0.22	0.47	ENSMUSG00000044083.12	Efcab8	hypo
chr5	137569365	137569673	1.97E-09	0.47	0.22	0.47	ENSMUSG00000029716.13	Tfr2	hypo
chr2	91932145	91932361	9.46E-04	0.21	0.1	0.48	ENSMUSG00000027239.14	Mdk	hypo
chr5	24424253	24424568	9.95E-06	0.21	0.1	0.48	ENSMUSG00000028969.10	Cdk5	hypo
chr8	34805387	34805601	4.77E-05	0.21	0.1	0.48	ENSMUSG00000031530.6	Dusp4	hypo
chr13	108449161	108449470	1.26E-03	0.23	0.11	0.48	ENSMUSG00000021699.17	Pde4d	hypo
chr15	81859772	81860120	2.40E-07	0.23	0.11	0.48	ENSMUSG00000048546.8	Tob2	hypo
chr4	126325632	126325910	1.08E-08	0.23	0.11	0.48	ENSMUSG00000028845.15	Tekt2	hypo
chr5	115272977	115273513	3.48E-12	0.48	0.23	0.48	ENSMUSG00000041740.16	Rnf10	hypo
chr1	150098850	150099281	5.74E-06	0.5	0.24	0.48	ENSMUSG00000032487.8	Ptgs2	hypo
chr5	114971177	114971582	1.37E-05	0.25	0.12	0.48	ENSMUSG00000029556.12	Hnf1a	hypo
chr15	79027117	79027413	7.09E-09	0.27	0.13	0.48	ENSMUSG00000096210.2	H1f0	hypo
chr5	119668649	119669219	1.17E-16	0.27	0.13	0.48	ENSMUSG00000018604.18	Tbx3	hypo
chr6	52192706	52193057	5.67E-06	0.27	0.13	0.48	ENSMUSG00000000942.10	Hoxa4	hypo
chr16	45492864	45493194	1.29E-33	0.83	0.4	0.48	ENSMUSG00000053182.11	Gm609	hypo
chr10	128803661	128803897	3.65E-06	0.29	0.14	0.48	ENSMUSG00000025354.5	Dnajc14	hypo
chr14	69766983	69767427	4.35E-08	0.31	0.15	0.48	ENSMUSG00000022074.6	Tnfrsf10b	hypo
chr1	21963353	21963572	1.71E-05	0.33	0.16	0.48	ENSMUSG00000028033.16	Kcnq5	hypo
chr2	152413447	152413676	2.12E-08	0.33	0.16	0.48	ENSMUSG00000032680.11	6820408C15Rik	hypo
chr13	108315593	108315852	7.29E-06	0.35	0.17	0.49	ENSMUSG00000021697.12	Depdc1b	hypo
chr16	23144408	23144676	7.67E-06	0.35	0.17	0.49	ENSMUSG00000022878.5	Adipoq	hypo
chr2	152413422	152413646	1.15E-09	0.37	0.18	0.49	ENSMUSG00000032680.11	6820408C15Rik	hypo
chr1	21963515	21963764	3.91E-05	0.41	0.2	0.49	ENSMUSG00000028033.16	Kcnq5	hypo
chr8	71701807	71702256	7.55E-06	0.45	0.22	0.49	ENSMUSG00000031803.8	B3gnt3	hypo
chr11	117827056	117827540	2.85E-06	0.3	0.15	0.50	ENSMUSG00000025574.13	Tk1	hypo
chr15	79027107	79027366	4.74E-08	0.24	0.12	0.50	ENSMUSG00000096210.2	H1f0	hypo
chr15	81859913	81860120	5.84E-03	0.22	0.11	0.50	ENSMUSG00000048546.8	Tob2	hypo
chr15	99716000	99716215	3.65E-06	0.34	0.17	0.50	ENSMUSG00000023019.12	Gpd1	hypo
chr1	182563271	182563479	1.27E-06	0.28	0.14	0.50	ENSMUSG00000038599.14	Capn8	hypo
chr2	35337469	35337731	1.19E-05	0.2	0.1	0.50	ENSMUSG00000026880.11	Stom	hypo
chr2	84775035	84775545	1.32E-04	0.24	0.12	0.50	ENSMUSG00000023224.12	Serping1	hypo
chr4	155962275	155962635	5.01E-18	0.38	0.19	0.50	ENSMUSG00000023571.4	C1qtnf12	hypo
chr6	142571877	142572247	1.52E-06	0.24	0.12	0.50	ENSMUSG00000030247.9	Kcnj8	hypo
chr7	46030559	46030948	9.30E-08	0.28	0.14	0.50	ENSMUSG00000030834.7	Abcc6	hypo
chr8	70634277	70634525	7.13E-04	0.26	0.13	0.50	ENSMUSG00000038508.7	Gdf15	hypo
chr9	21835213	21835484	2.25E-07	0.34	0.17	0.50	ENSMUSG00000047822.8	Angptl8	hypo
chr9	119052490	119052882	2.60E-06	0.22	0.11	0.50	ENSMUSG00000038775.14	Vill	hypo
chrX	100594335	100595035	2.60E-05	0.42	0.21	0.50	ENSMUSG00000044359.1	P2ry4	hypo
chr12	116404418	116405117	2.31E-83	0.35	0.7	2.00	ENSMUSG00000042029.7	Ncapg2	hyper
chr13	4572388	4572603	8.76E-10	0.4	0.8	2.00	ENSMUSG00000021207.9	Akr1c21	hyper
chr13	41114128	41114350	8.00E-11	0.17	0.34	2.00	ENSMUSG00000038651.15	Sycp2l	hyper
chr17	34882079	34882297	1.74E-03	0.11	0.22	2.00	ENSMUSG00000092511.7	Gm20547	hyper
chr19	24557842	24558093	1.30E-03	0.11	0.22	2.00	ENSMUSG00000024867.14	Pip5k1b	hyper
chr1	36539173	36539464	4.39E-04	0.11	0.22	2.00	ENSMUSG00000109510.1	Gm42417	hyper
chr1	89932576	89932838	6.07E-06	0.1	0.2	2.00	ENSMUSG00000034486.8	Gbx2	hyper
chr2	19199184	19199753	3.38E-14	0.12	0.24	2.00	ENSMUSG00000037683.14	Armc3	hyper
chr2	58565346	58565694	7.63E-04	0.1	0.2	2.00	ENSMUSG00000026839.17	Upp2	hyper
chr3	96575719	96576014	3.91E-07	0.24	0.48	2.00	ENSMUSG00000087610.1	Gm16253	hyper
chr4	96591063	96592057	1.11E-19	0.16	0.32	2.00	ENSMUSG00000015224.10	Cyp2j9	hyper
chr8	9977101	9977325	1.62E-09	0.12	0.24	2.00	ENSMUSG00000040396.12	Abhd13	hyper
chr8	117673295	117673904	4.83E-24	0.21	0.42	2.00	ENSMUSG00000034308.13	Sdr42e1	hyper
chrX	7572603	7572852	3.69E-07	0.2	0.4	2.00	ENSMUSG00000039521.13	Foxp3	hyper
chr19	44201404	44201606	2.75E-15	0.39	0.79	2.03	ENSMUSG00000025202.8	Scd3	hyper
chr16	45222638	45223016	1.93E-14	0.37	0.75	2.03	ENSMUSG00000052013.15	Btla	hyper
chr12	116404348	116405117	3.05E-85	0.35	0.71	2.03	ENSMUSG00000042029.7	Ncapg2	hyper
chr8	117673333	117673967	1.66E-27	0.22	0.45	2.05	ENSMUSG00000034308.13	Sdr42e1	hyper
chr8	116504286	116504509	1.70E-04	0.17	0.35	2.06	ENSMUSG00000034467.7	Dynlrb2	hyper
chr13	41114183	41114395	5.57E-11	0.16	0.33	2.06	ENSMUSG00000038651.15	Sycp2l	hyper
chr19	44555500	44555864	9.23E-10	0.16	0.33	2.06	ENSMUSG00000025204.10	Ndufb8	hyper
chr6	137170171	137170658	2.53E-06	0.16	0.33	2.06	ENSMUSG00000030222.13	Rerg	hyper
chr2	93640709	93640986	4.28E-07	0.15	0.31	2.07	ENSMUSG00000040310.12	Alx4	hyper
chr4	95557345	95557624	1.07E-08	0.15	0.31	2.07	ENSMUSG00000028573.18	Fggy	hyper
chr2	104069871	104070086	1.23E-05	0.14	0.29	2.07	ENSMUSG00000075006.2	Gm10799	hyper
chr2	151996096	151996343	4.66E-05	0.14	0.29	2.07	ENSMUSG00000027463.14	Slc52a3	hyper
chr6	137170120	137170500	1.99E-05	0.14	0.29	2.07	ENSMUSG00000030222.13	Rerg	hyper
chr12	84316155	84316396	6.21E-06	0.13	0.27	2.08	ENSMUSG00000042472.11	Zfp410	hyper
chr5	8998085	8998356	1.05E-03	0.13	0.27	2.08	ENSMUSG00000003623.4	Crot	hyper
chr17	84682781	84683083	3.49E-07	0.12	0.25	2.08	ENSMUSG00000040505.13	Abcg5	hyper

chr4	117124742	117125152	2.46E-06	0.12	0.25	2.08	ENSMUSG00000047671.8	Tctex1d4	hyper
chr19	47537246	47538129	2.14E-30	0.23	0.48	2.09	ENSMUSG00000042694.17	Stn1	hyper
chr11	90030229	90030578	9.16E-05	0.11	0.23	2.09	ENSMUSG00000069763.3	Tmem100	hyper
chr1	171360599	171360834	2.03E-06	0.11	0.23	2.09	ENSMUSG00000045259.4	Klhdc9	hyper
chr8	14880198	14880428	4.60E-06	0.11	0.23	2.09	ENSMUSG00000026317.7	Cln8	hyper
chrX	50944388	50944680	2.27E-04	0.22	0.46	2.09	ENSMUSG00000036131.12	Frmd7	hyper
chr11	80809665	80810025	7.13E-06	0.1	0.21	2.10	ENSMUSG00000035413.8	Tmem98	hyper
chr16	92300586	92300900	1.32E-10	0.2	0.42	2.10	ENSMUSG00000051989.11	Smim11	hyper
chr1	36539254	36539529	3.78E-04	0.1	0.21	2.10	ENSMUSG00000109510.1	Gm42417	hyper
chr12	116404525	116405117	8.15E-49	0.29	0.61	2.10	ENSMUSG00000042029.7	Ncapg2	hyper
chr17	88791516	88792240	1.25E-31	0.17	0.36	2.12	ENSMUSG00000024107.7	Lhcgr	hyper
chr17	88791684	88792329	3.59E-31	0.17	0.36	2.12	ENSMUSG00000024107.7	Lhcgr	hyper
chr11	93994509	93994886	2.40E-05	0.16	0.34	2.13	ENSMUSG00000020859.16	Spag9	hyper
chr6	34383834	34384111	5.60E-06	0.16	0.34	2.13	ENSMUSG00000061758.13	Akr1b10	hyper
chr4	95557422	95557624	5.38E-09	0.15	0.32	2.13	ENSMUSG00000028573.18	Fggy	hyper
chr16	32165571	32165875	8.34E-07	0.13	0.28	2.15	ENSMUSG00000052384.14	Nrros	hyper
chr18	74267360	74267607	3.35E-04	0.26	0.56	2.15	ENSMUSG00000024561.11	Mbd1	hyper
chr16	92300633	92301066	2.48E-08	0.12	0.26	2.17	ENSMUSG00000051989.11	Smim11	hyper
chr8	71568540	71568794	6.97E-04	0.12	0.26	2.17	ENSMUSG00000034829.8	Nxnl1	hyper
chr8	71568540	71568794	6.97E-04	0.12	0.26	2.17	ENSMUSG00000031808.7	Slc27a1	hyper
chr16	33379503	33379765	2.86E-05	0.11	0.24	2.18	ENSMUSG00000022811.17	Zfp148	hyper
chr17	35438882	35439150	1.24E-03	0.11	0.24	2.18	ENSMUSG00000060550.16	H2-Q7	hyper
chr2	120609768	120610288	6.06E-06	0.11	0.24	2.18	ENSMUSG00000027286.16	Lrrc57	hyper
chr12	35536091	35536427	7.36E-12	0.32	0.7	2.19	ENSMUSG00000019256.17	Ahr	hyper
chrX	107816553	107816813	8.95E-06	0.21	0.46	2.19	ENSMUSG00000031242.7	2610002M06Rik	hyper
chr17	15564777	15565098	3.74E-05	0.1	0.22	2.20	ENSMUSG00000051977.16	Prdm9	hyper
chr8	63952106	63952351	5.03E-10	0.1	0.22	2.20	ENSMUSG00000094443.2	Sgo2b	hyper
chr17	23740741	23741048	3.18E-05	0.09	0.2	2.22	ENSMUSG00000023909.4	Paqr4	hyper
chr12	116404531	116405117	2.50E-45	0.26	0.58	2.23	ENSMUSG00000042029.7	Ncapg2	hyper
chr13	104791561	104791855	2.32E-14	0.3	0.67	2.23	ENSMUSG00000021716.14	Srek1ip1	hyper
chr19	44028988	44029259	1.20E-11	0.29	0.65	2.24	ENSMUSG00000025197.9	Cyp2c23	hyper
chr13	117206172	117206577	1.09E-17	0.35	0.79	2.26	ENSMUSG00000021728.8	Emb	hyper
chr17	84682787	84683083	1.71E-07	0.11	0.25	2.27	ENSMUSG00000040505.13	Abcg5	hyper
chr4	117124899	117125152	2.83E-07	0.11	0.25	2.27	ENSMUSG00000047671.8	Tctex1d4	hyper
chr9	121277927	121278270	1.17E-24	0.25	0.57	2.28	ENSMUSG00000040936.15	Ulk4	hyper
chrX	167209531	167210712	7.61E-17	0.17	0.39	2.29	ENSMUSG00000049775.16	Tmsb4x	hyper
chr15	76818346	76818710	1.60E-05	0.1	0.23	2.30	ENSMUSG00000033697.16	Arhgap39	hyper
chr1	156205143	156205556	9.39E-08	0.09	0.21	2.33	ENSMUSG00000015484.3	Fam163a	hyper
chr14	66344167	66344402	2.62E-06	0.11	0.26	2.36	ENSMUSG00000022044.14	Stmn4	hyper
chr12	84316172	84316396	3.33E-06	0.1	0.24	2.40	ENSMUSG00000042472.11	Zfp410	hyper
chr12	116404587	116405117	4.44E-38	0.22	0.53	2.41	ENSMUSG00000042029.7	Ncapg2	hyper
chr17	47611912	47612447	1.16E-18	0.12	0.29	2.42	ENSMUSG00000023988.8	Bysl	hyper
chr9	40531490	40531748	2.14E-06	0.09	0.22	2.44	ENSMUSG00000040111.17	Gramd1b	hyper
chr17	83849663	83849956	1.05E-05	0.28	0.69	2.46	ENSMUSG00000000673.9	Haao	hyper
chr19	3913106	3913695	6.92E-12	0.12	0.3	2.50	ENSMUSG00000059734.7	Ndufs8	hyper
chr1	180568311	180568800	6.95E-10	0.08	0.2	2.50	ENSMUSG00000026496.11	Parp1	hyper
chr2	15056358	15056803	1.81E-05	0.1	0.25	2.50	ENSMUSG00000026707.15	Nsun6	hyper
chr10	85927332	85927623	2.14E-04	0.11	0.28	2.55	ENSMUSG00000085111.3	Ascl4	hyper
chr17	34398029	34399607	5.43E-35	0.08	0.21	2.63	ENSMUSG00000057246.16	BC051142	hyper
chr5	138171641	138171871	2.15E-26	0.08	0.21	2.63	ENSMUSG00000019518.11	Ap4m1	hyper
chr7	38528982	38529261	5.60E-08	0.16	0.43	2.69	ENSMUSG00000060565.5	Gm5591	hyper
chr5	135632354	135632563	2.14E-07	0.1	0.27	2.70	ENSMUSG00000039917.10	Rhbdd2	hyper
chr11	104607147	104607369	4.00E-09	0.16	0.46	2.88	ENSMUSG00000020689.4	Itgb3	hyper
chr19	47854231	47854823	1.42E-10	0.09	0.27	3.00	ENSMUSG00000025068.8	Gsto1	hyper
chr8	95350967	95352156	5.55E-90	0.15	0.47	3.13	ENSMUSG00000031790.8	Mmp15	hyper
chr1	9544527	9545030	4.57E-11	0.08	0.26	3.25	ENSMUSG00000061024.8	Rrs1	hyper
chr17	34398117	34399607	6.52E-44	0.06	0.21	3.50	ENSMUSG00000057246.16	BC051142	hyper
chr6	142702557	142702878	1.17E-10	0.09	0.32	3.56	ENSMUSG00000030249.15	Abcc9	hyper
chr15	53902254	53902928	1.32E-29	0.06	0.22	3.67	ENSMUSG00000058656.13	Samd12	hyper
chr5	137601643	137602086	3.07E-28	0.06	0.22	3.67	ENSMUSG00000037221.13	Mospd3	hyper
chr2	38927370	38927777	2.10E-19	0.11	0.41	3.73	ENSMUSG00000063972.13	Nr6a1	hyper
chr8	95351337	95352156	4.70E-89	0.1	0.42	4.20	ENSMUSG00000031790.8	Mmp15	hyper
chr8	126945603	126945848	8.32E-79	0.06	0.28	4.67	ENSMUSG00000093904.2	Tomm20	hyper

Supplementary Table S6. The methylation profiles of the control group and high-dose PFOS group, at the proximal promoter (2kb) of protein-coding genes.

Chr	Start	End	p-value	Ave CpG methylation (Ctrl)	Ave CpG methylation rate (high dose PFOS)	high dose PFOS/Ctrl	Gene ID	Gene name	hypo/hyper
chr2	177839863	177840562	9.95E-69	0.24	0.01	0.042	ENSMUSG00000095362.7	Gm14325	hypo
chr13	4608557	4609466	5.24E-119	0.49	0.04	0.082	ENSMUSG00000045410.18	Akr1e1	hypo
chr1	171344655	171345244	5.20E-41	0.45	0.11	0.24	ENSMUSG00000006412.10	Pfdn2	hypo
chr11	69948988	69949374	3.06E-12	0.2	0.05	0.25	ENSMUSG00000018566.14	Slc2a4	hypo
chr12	71136476	71136730	1.71E-39	0.28	0.07	0.25	ENSMUSG00000021079.16	Timm9	hypo
chr12	71136476	71136730	1.71E-39	0.28	0.07	0.25	ENSMUSG00000034601.17	2700049A03Rik	hypo
chr2	177464490	177465439	1.40E-43	0.36	0.09	0.25	ENSMUSG00000078866.10	Zfp970	hypo
chr7	89405932	89406135	1.47E-06	0.22	0.06	0.27	ENSMUSG00000039428.11	Tmem135	hypo
chr11	60535838	60536402	3.81E-27	0.76	0.21	0.28	ENSMUSG00000042650.3	Alkbh5	hypo
chr2	177497959	177498716	4.84E-57	0.41	0.13	0.32	ENSMUSG00000094786.1	Gm14403	hypo
chr1	160905731	160906088	4.52E-06	0.27	0.09	0.33	ENSMUSG00000040423.11	Rc3h1	hypo
chr4	147305564	147305821	5.08E-10	0.74	0.26	0.35	ENSMUSG00000078498.5	Zfp988	hypo
chr7	25072934	25073195	7.59E-06	0.31	0.11	0.35	ENSMUSG00000003378.9	Grik5	hypo
chr1	172376530	172378006	4.22E-103	0.28	0.1	0.36	ENSMUSG00000050229.3	Pigm	hypo
chr3	139205275	139205625	4.41E-18	0.25	0.09	0.36	ENSMUSG00000047940.13	Stpg2	hypo
chr12	74316457	74317700	8.89E-32	0.38	0.14	0.37	ENSMUSG00000079061.3	Gm11042	hypo
chr2	9880672	9881141	6.83E-10	0.21	0.08	0.38	ENSMUSG00000025783.2	4930412O13Rik	hypo
chr6	124808293	124808660	5.78E-04	0.21	0.08	0.38	ENSMUSG00000038451.13	Spsb2	hypo
chr8	70634224	70634640	2.23E-05	0.21	0.08	0.38	ENSMUSG00000038508.7	Gdf15	hypo
chr2	177397987	177398548	2.35E-24	0.52	0.2	0.38	ENSMUSG00000078867.9	Gm14418	hypo
chr11	4637216	4637482	3.93E-07	0.23	0.09	0.39	ENSMUSG00000020412.16	Ascc2	hypo
chr2	27675937	27676240	1.37E-10	0.28	0.11	0.39	ENSMUSG00000015846.15	Rxra	hypo
chr7	12998093	12998409	3.22E-05	0.28	0.11	0.39	ENSMUSG00000030382.15	Slc27a5	hypo
chr7	12998093	12998409	3.22E-05	0.28	0.11	0.39	ENSMUSG00000005566.14	Trim28	hypo
chr13	98694698	98694925	3.32E-09	0.2	0.08	0.4	ENSMUSG00000052485.7	Tmem171	hypo
chr4	107685422	107685624	5.15E-09	0.8	0.32	0.4	ENSMUSG00000028610.16	Dmrtb1	hypo
chr9	45116443	45117055	7.74E-18	0.45	0.18	0.4	ENSMUSG00000070304.12	Scn2b	hypo
chr15	54953815	54954239	5.94E-09	0.3	0.12	0.4	ENSMUSG00000022425.16	Enpp2	hypo
chr9	45054629	45055133	1.73E-14	0.27	0.11	0.41	ENSMUSG00000070305.10	Mpzl3	hypo
chr18	9210611	9210828	1.38E-03	0.22	0.09	0.41	ENSMUSG00000036904.6	Fzd8	hypo
chr8	85300368	85300906	1.26E-23	0.44	0.18	0.41	ENSMUSG00000031696.9	Vps35	hypo
chr3	10331475	10331899	2.88E-06	0.34	0.14	0.41	ENSMUSG00000027531.16	Impa1	hypo
chr5	115558087	115558459	1.47E-06	0.29	0.12	0.41	ENSMUSG00000067274.10	Rplp0	hypo
chr18	46728674	46728897	1.23E-03	0.24	0.1	0.42	ENSMUSG00000033022.8	Cdo1	hypo
chr2	69342810	69343210	2.06E-04	0.24	0.1	0.42	ENSMUSG00000027048.15	Abcb11	hypo
chr2	172369589	172370003	5.54E-07	0.26	0.11	0.42	ENSMUSG00000027498.14	Cstf1	hypo
chr18	36726735	36727086	9.31E-06	0.33	0.14	0.42	ENSMUSG00000051439.7	Cd14	hypo
chr4	145248187	145248541	9.48E-25	0.87	0.37	0.43	ENSMUSG00000028599.10	Tnfrsf1b	hypo
chr3	139205130	139205625	6.79E-19	0.28	0.12	0.43	ENSMUSG00000047940.13	Stpg2	hypo
chr11	48872825	48873222	8.03E-05	0.21	0.09	0.43	ENSMUSG00000046879.7	Irgm1	hypo
chr11	118290550	118290974	6.83E-05	0.21	0.09	0.43	ENSMUSG00000033909.17	Usp36	hypo
chr8	126971423	126971685	1.74E-06	0.21	0.09	0.43	ENSMUSG00000033931.9	Rbm34	hypo
chr16	23144408	23144676	3.95E-05	0.35	0.15	0.43	ENSMUSG00000022878.5	Adipoq	hypo
chr2	25095290	25095605	3.71E-08	0.23	0.1	0.43	ENSMUSG00000036805.13	Noxa1	hypo
chr6	142571742	142572247	1.45E-06	0.23	0.1	0.43	ENSMUSG00000030247.9	Kcnj8	hypo
chr11	96363843	96364095	5.87E-04	0.32	0.14	0.44	ENSMUSG00000018973.2	Hoxb1	hypo
chr18	31921963	31922217	1.02E-06	0.32	0.14	0.44	ENSMUSG00000024395.9	Lims2	hypo
chr16	18424737	18425000	1.41E-05	0.25	0.11	0.44	ENSMUSG00000075704.16	Txnrd2	hypo
chr8	121113947	121114214	1.30E-05	0.25	0.11	0.44	ENSMUSG00000046714.7	Foxc2	hypo
chr2	10153056	10153536	6.31E-11	0.34	0.15	0.44	ENSMUSG00000025780.7	Itih5	hypo
chr2	177397929	177398548	4.66E-22	0.52	0.23	0.44	ENSMUSG00000078867.9	Gm14418	hypo
chr2	177759244	177759723	9.82E-23	0.52	0.23	0.44	ENSMUSG00000078864.10	Gm14322	hypo
chr11	99231084	99231451	2.25E-07	0.27	0.12	0.44	ENSMUSG00000037935.16	Smarce1	hypo
chr13	116311442	116312713	4.18E-13	0.27	0.12	0.44	ENSMUSG00000042258.13	Isl1	hypo
chr11	59473447	59473813	3.54E-14	0.9	0.4	0.44	ENSMUSG00000054519.8	Zfp867	hypo
chr5	146832469	146832830	4.19E-05	0.29	0.13	0.45	ENSMUSG00000041453.12	Rpl21	hypo
chr2	35337469	35337731	1.55E-05	0.2	0.09	0.45	ENSMUSG00000026880.11	Stom	hypo
chr5	115272996	115273341	5.64E-07	0.4	0.18	0.45	ENSMUSG00000041740.16	Rnf10	hypo
chr17	84683293	84683818	1.80E-10	0.31	0.14	0.45	ENSMUSG00000040505.13	Abcg5	hypo
chr4	43560320	43560721	5.54E-05	0.31	0.14	0.45	ENSMUSG00000028466.15	Creb3	hypo
chr13	51170385	51170663	1.21E-06	0.33	0.15	0.45	ENSMUSG00000021396.5	Nxnl2	hypo
chr4	147850242	147850532	1.70E-09	0.66	0.3	0.45	ENSMUSG00000059423.13	Zfp933	hypo
chr16	45408803	45409197	1.53E-07	0.22	0.1	0.45	ENSMUSG00000022661.14	Cd200	hypo
chr2	10153014	10153536	1.19E-11	0.35	0.16	0.46	ENSMUSG00000025780.7	Itih5	hypo
chr2	25395476	25395860	3.48E-07	0.24	0.11	0.46	ENSMUSG00000015085.8	Entpd2	hypo
chr11	118420348	118420753	4.86E-06	0.37	0.17	0.46	ENSMUSG00000025575.14	Cant1	hypo
chr10	69149347	69149559	9.49E-06	0.39	0.18	0.46	ENSMUSG00000019944.14	Rhobtb1	hypo
chr13	116310793	116312713	2.23E-14	0.26	0.12	0.46	ENSMUSG00000042258.13	Isl1	hypo
chr16	90737018	90737281	1.59E-04	0.26	0.12	0.46	ENSMUSG00000039956.9	Mrap	hypo
chr3	40948779	40949044	1.21E-05	0.26	0.12	0.46	ENSMUSG00000025762.16	Larp1b	hypo
chr4	99930381	99930855	1.85E-07	0.26	0.12	0.46	ENSMUSG00000028549.17	Itgb3bp	hypo
chr6	124814370	124815085	1.12E-07	0.26	0.12	0.46	ENSMUSG00000023456.16	Tpi1	hypo
chr11	62878685	62879125	1.91E-08	0.41	0.19	0.46	ENSMUSG00000014177.10	Tvp23b	hypo
chr7	16982779	16983061	2.43E-05	0.28	0.13	0.46	ENSMUSG00000108348.2	Gm42372	hypo
chrX	7681122	7681425	2.88E-03	0.28	0.13	0.46	ENSMUSG00000031147.8	Magix	hypo
chr8	83743009	83743408	2.18E-29	0.9	0.42	0.47	ENSMUSG00000002885.14	Adgre5	hypo
chr13	43481810	43482297	1.08E-07	0.3	0.14	0.47	ENSMUSG00000038546.9	Ranbp9	hypo
chr8	13060772	13061054	4.57E-05	0.3	0.14	0.47	ENSMUSG00000031445.5	Proz	hypo
chr2	28086142	28086474	3.72E-27	0.77	0.36	0.47	ENSMUSG00000026835.15	Fcnb	hypo
chr18	56400005	56400466	8.40E-07	0.32	0.15	0.47	ENSMUSG00000001700.10	Gramd3	hypo
chr7	112158665	112159102	1.19E-10	0.32	0.15	0.47	ENSMUSG00000030772.6	Dkk3	hypo
chr8	124570030	124570471	1.70E-07	0.32	0.15	0.47	ENSMUSG00000031980.10	Agt	hypo
chr10	117224799	117225347	1.97E-19	0.51	0.24	0.47	ENSMUSG00000020171.8	Yeats4	hypo
chr18	56399955	56400449	9.19E-08	0.34	0.16	0.47	ENSMUSG00000001700.10	Gramd3	hypo
chr3	30549537	30549762	1.07E-05	0.34	0.16	0.47	ENSMUSG00000027684.16	Mecom	hypo
chr15	31369615	31369930	3.26E-09	0.53	0.25	0.47	ENSMUSG00000022237.17	Ankrd33b	hypo
chr1	93160853	93161093	1.09E-06	0.36	0.17	0.47	ENSMUSG00000034159.12	Mab21l4	hypo
chr4	128618506	128618831	1.18E-07	0.36	0.17	0.47	ENSMUSG00000062545.4	Tlr12	hypo
chr2	70472562	70473429	1.87E-13	0.38	0.18	0.47	ENSMUSG00000075304.2	Sp5	hypo
chr2	167687563	167688014	6.35E-10	0.38	0.18	0.47	ENSMUSG00000056501.3	Cebpb	hypo
chr12	74316418	74317700	3.26E-23	0.4	0.19	0.48	ENSMUSG00000079061.3	Gm11042	hypo
chr13	23806472	23806690	3.14E-08	0.4	0.19	0.48	ENSMUSG00000036110.14	Slc17a2	hypo
chr4	148158854	148159156	2.08E-10	0.42	0.2	0.48	ENSMUSG00000041556.8	Fbxo2	hypo
chr8	11313514	11313721	5.28E-04	0.21	0.1	0.48	ENSMUSG00000031502.11	Col4a1	hypo
chr11	70615147	70615594	1.37E-12	0.23	0.11	0.48	ENSMUSG00000087279.10	4930544D05Rik	hypo
chr18	36678189	36678590	3.81E-03	0.23	0.11	0.48	ENSMUSG00000033272.12	Slc35a4	hypo
chr19	43986693	43986918	1.63E-04	0.23	0.11	0.48	ENSMUSG00000025196.4	Cpn1	hypo
chr8	71525830	71526289	5.80E-04	0.23	0.11	0.48	ENSMUSG00000108900.1	Ccdc194	hypo
chr8	121113835	121114214	2.35E-06	0.23	0.11	0.48	ENSMUSG00000046714.7	Foxc2	hypo
chr1	88509967	88510180	5.12E-06	0.69	0.33	0.48	ENSMUSG00000062310.7	Glrp1	hypo
chr8	83742835	83743193	4.68E-31	0.94	0.45	0.48	ENSMUSG00000002885.14	Adgre5	hypo
chr9	45116293	45117055	1.52E-15	0.48	0.23	0.48	ENSMUSG00000070304.12	Scn2b	hypo

chr10	77528714	77528994	8.02E-04	0.25	0.12	0.48	ENSMUSG00000000290.13	Itgb2	hypo
chr10	81168262	81168559	2.72E-04	0.25	0.12	0.48	ENSMUSG00000004934.14	Pias4	hypo
chr2	167691109	167691413	1.55E-06	0.25	0.12	0.48	ENSMUSG00000090213.1	Tmem189	hypo
chr4	147940280	147940606	8.53E-04	0.25	0.12	0.48	ENSMUSG00000044496.6	2510039O18Rik	hypo
chr17	84683484	84684136	1.03E-07	0.27	0.13	0.48	ENSMUSG00000040505.13	Abcg5	hypo
chr5	77002727	77003004	1.53E-24	0.56	0.27	0.48	ENSMUSG00000063820.11	Arl9	hypo
chr13	23866206	23866786	3.58E-07	0.29	0.14	0.48	ENSMUSG00000021335.13	Slc17a1	hypo
chr2	167691140	167691521	1.05E-05	0.29	0.14	0.48	ENSMUSG00000090213.1	Tmem189	hypo
chr8	13060741	13060952	1.48E-04	0.29	0.14	0.48	ENSMUSG00000031445.5	Proz	hypo
chr3	146570239	146570862	4.20E-08	0.31	0.15	0.48	ENSMUSG00000028186.14	Uox	hypo
chr4	128618361	128618831	1.71E-07	0.31	0.15	0.48	ENSMUSG00000062545.4	Tlr12	hypo
chr7	25897349	25897763	2.47E-14	0.62	0.3	0.48	ENSMUSG00000030483.14	Cyp2b10	hypo
chr9	37527166	37527374	2.81E-05	0.31	0.15	0.48	ENSMUSG00000001946.14	Esam	hypo
chr7	103521705	103522133	2.63E-07	0.64	0.31	0.48	ENSMUSG00000096584.2	Olfr611	hypo
chr3	146570146	146570767	2.14E-09	0.33	0.16	0.48	ENSMUSG00000028186.14	Uox	hypo
chr9	45054542	45055133	1.07E-14	0.33	0.16	0.48	ENSMUSG00000070305.10	Mpzl3	hypo
chr13	43305358	43305562	1.37E-05	0.35	0.17	0.49	ENSMUSG00000051335.6	Gfod1	hypo
chr19	46046653	46046910	9.43E-05	0.35	0.17	0.49	ENSMUSG00000025223.15	Ldb1	hypo
chr7	112158714	112159120	1.10E-11	0.35	0.17	0.49	ENSMUSG00000030772.6	Dkk3	hypo
chr13	43305379	43305654	3.52E-06	0.37	0.18	0.49	ENSMUSG00000051335.6	Gfod1	hypo
chr6	52192828	52193100	8.16E-06	0.37	0.18	0.49	ENSMUSG00000000942.10	Hoxa4	hypo
chr11	110096834	110097120	3.35E-06	0.39	0.19	0.49	ENSMUSG00000041828.15	Abca8a	hypo
chr4	43560313	43560661	4.16E-06	0.39	0.19	0.49	ENSMUSG00000028466.15	Creb3	hypo
chr13	51170305	51170597	1.44E-08	0.41	0.2	0.49	ENSMUSG00000021396.5	Nxnl2	hypo
chr6	124814542	124815085	1.24E-06	0.41	0.2	0.49	ENSMUSG00000023456.16	Tpi1	hypo
chrX	99974727	99975034	4.11E-04	0.41	0.2	0.49	ENSMUSG00000059327.9	Eda	hypo
chr15	78468205	78468695	3.17E-10	0.45	0.22	0.49	ENSMUSG00000016942.6	Tmprss6	hypo
chr5	77002780	77003153	1.54E-27	0.57	0.28	0.49	ENSMUSG00000063820.11	Arl9	hypo
chr1	88509909	88510136	4.74E-05	0.61	0.3	0.49	ENSMUSG00000062310.7	Glrp1	hypo
chr7	25897362	25897787	1.02E-13	0.61	0.3	0.49	ENSMUSG00000030483.14	Cyp2b10	hypo
chr5	121661905	121662113	1.86E-08	0.63	0.31	0.49	ENSMUSG00000029456.12	Acad10	hypo
chr10	59403520	59403735	5.78E-05	0.24	0.12	0.5	ENSMUSG00000009646.13	Pla2g12b	hypo
chr10	100588368	100588972	4.94E-11	0.36	0.18	0.5	ENSMUSG00000056912.12	1700017N19Rik	hypo
chr10	117224660	117225273	2.86E-12	0.4	0.2	0.5	ENSMUSG00000020171.8	Yeats4	hypo
chr12	111486199	111486407	2.83E-07	0.26	0.13	0.5	ENSMUSG00000010529.6	Gm266	hypo
chr13	23866230	23866805	1.81E-07	0.34	0.17	0.5	ENSMUSG00000021335.13	Slc17a1	hypo
chr14	121506398	121506742	2.16E-04	0.3	0.15	0.5	ENSMUSG00000025557.10	Slc15a1	hypo
chr17	46888614	46888990	7.76E-06	0.28	0.14	0.5	ENSMUSG00000036430.8	Tbcc	hypo
chr1	90202920	90203151	2.48E-05	0.3	0.15	0.5	ENSMUSG00000044337.5	Ackr3	hypo
chr1	160978515	160978793	4.08E-11	0.58	0.29	0.5	ENSMUSG00000026715.12	Serpinc1	hypo
chr2	119324146	119324411	2.79E-05	0.32	0.16	0.5	ENSMUSG00000027314.6	Dll4	hypo
chr2	164831049	164831329	8.41E-18	0.68	0.34	0.5	ENSMUSG00000017760.16	Ctsa	hypo
chr2	167687559	167688014	8.29E-10	0.38	0.19	0.5	ENSMUSG00000056501.3	Cebpb	hypo
chr3	10302732	10303065	8.88E-04	0.3	0.15	0.5	ENSMUSG00000027530.15	Fabp12	hypo
chr3	29081784	29082003	4.15E-06	0.28	0.14	0.5	ENSMUSG00000063600.14	Egfem1	hypo
chr4	107801468	107801708	1.80E-03	0.28	0.14	0.5	ENSMUSG00000028613.15	Lrp8	hypo
chr4	148158842	148159103	6.52E-12	0.48	0.24	0.5	ENSMUSG00000041556.8	Fbxo2	hypo
chr4	155962260	155963002	1.14E-28	0.4	0.2	0.5	ENSMUSG00000023571.4	C1qtnf12	hypo
chr6	52205471	52205688	7.94E-07	0.26	0.13	0.5	ENSMUSG00000038253.6	Hoxa5	hypo
chr7	44815019	44815370	5.65E-06	0.26	0.13	0.5	ENSMUSG00000109511.1	Nup62	hypo
chr7	44815019	44815370	5.65E-06	0.26	0.13	0.5	ENSMUSG00000074141.13	Il4i1	hypo
chr7	84606947	84607221	1.42E-05	0.28	0.14	0.5	ENSMUSG00000030630.16	Fah	hypo
chr7	122670423	122670890	1.80E-07	0.24	0.12	0.5	ENSMUSG00000066189.9	Cacng3	hypo
chr8	106602848	106603100	4.28E-06	0.2	0.1	0.5	ENSMUSG00000000303.12	Cdh1	hypo
chr9	21835479	21835791	7.61E-09	0.34	0.17	0.5	ENSMUSG00000047822.8	Angptl8	hypo
chr9	45116699	45117055	5.11E-09	0.4	0.2	0.5	ENSMUSG00000070304.12	Scn2b	hypo
chr10	110918878	110919101	1.07E-04	0.11	0.22	2	ENSMUSG00000020186.7	Csrp2	hyper
chr11	96291248	96291703	1.91E-08	0.15	0.3	2	ENSMUSG00000000690.5	Hoxb6	hyper
chr12	84316172	84316435	2.80E-05	0.14	0.28	2	ENSMUSG00000042472.11	Zfp410	hyper
chr12	84409311	84409677	8.25E-06	0.15	0.3	2	ENSMUSG00000021236.16	Entpd5	hyper
chr12	112586990	112587312	1.78E-05	0.16	0.32	2	ENSMUSG00000037679.9	Inf2	hyper
chr15	9139649	9139878	1.82E-04	0.16	0.32	2	ENSMUSG00000039704.7	Lmbrd2	hyper
chr15	81859950	81860178	5.18E-04	0.17	0.34	2	ENSMUSG00000048546.8	Tob2	hyper
chr16	4881811	4882188	7.19E-06	0.2	0.4	2	ENSMUSG00000039568.6	Ubald1	hyper
chr16	20496850	20497763	1.10E-38	0.22	0.44	2	ENSMUSG00000003235.13	Eif2b5	hyper
chr16	96144677	96144883	4.13E-05	0.15	0.3	2	ENSMUSG00000023147.17	Get1	hyper
chr19	44555548	44555864	1.01E-09	0.2	0.4	2	ENSMUSG00000025204.10	Ndufb8	hyper
chr19	47537246	47537850	7.89E-08	0.1	0.2	2	ENSMUSG00000042694.17	Stn1	hyper
chr1	74196360	74196782	3.08E-05	0.13	0.26	2	ENSMUSG00000048480.5	Cxcr1	hyper
chr1	171417646	171417989	5.83E-13	0.23	0.46	2	ENSMUSG00000103711.1	Tstd1	hyper
chr4	127970699	127971095	2.87E-09	0.2	0.4	2	ENSMUSG00000028813.2	CK137956	hyper
chr5	120887553	120887908	9.11E-05	0.14	0.28	2	ENSMUSG00000066861.14	Oas1g	hyper
chr5	123182735	123183054	2.66E-07	0.19	0.38	2	ENSMUSG00000029445.13	Hpd	hyper
chr7	7374247	7374824	8.01E-20	0.2	0.4	2	ENSMUSG00000110139.1	Gm45783	hyper
chr7	45211968	45212174	3.04E-04	0.12	0.24	2	ENSMUSG00000030792.8	Dkkl1	hyper
chr8	27042155	27042377	1.07E-06	0.3	0.6	2	ENSMUSG00000031485.15	Plpbp	hyper
chr8	83741459	83742180	1.35E-27	0.2	0.4	2	ENSMUSG00000002885.14	Adgre5	hyper
chr8	117095469	117095683	8.61E-05	0.15	0.3	2	ENSMUSG00000031845.15	Bco1	hyper
chr9	48481179	48481636	1.44E-13	0.18	0.36	2	ENSMUSG00000032026.7	Rexo2	hyper
chr13	104791561	104791870	2.84E-21	0.4	0.81	2.03	ENSMUSG00000021716.14	Srek1ip1	hyper
chr12	116404725	116404981	1.40E-20	0.35	0.71	2.03	ENSMUSG00000042029.7	Ncapg2	hyper
chr6	78393227	78393889	4.12E-06	0.32	0.65	2.03	ENSMUSG00000023140.4	Reg2	hyper
chr7	98146089	98146384	4.54E-10	0.32	0.65	2.03	ENSMUSG00000074006.3	Omp	hyper
chr12	110887964	110888219	1.83E-05	0.23	0.47	2.04	ENSMUSG00000021275.16	Tecpr2	hyper
chr12	116404760	116405071	1.23E-14	0.23	0.47	2.04	ENSMUSG00000042029.7	Ncapg2	hyper
chr17	43357745	43358582	5.81E-13	0.22	0.45	2.05	ENSMUSG00000056492.6	Adgrf5	hyper
chr7	27688344	27688710	1.49E-11	0.2	0.41	2.05	ENSMUSG00000057093.14	Zfp607b	hyper
chr13	117206112	117206577	3.20E-19	0.39	0.8	2.05	ENSMUSG00000021728.8	Emb	hyper
chrX	7572606	7572852	3.78E-05	0.18	0.37	2.06	ENSMUSG00000039521.13	Foxp3	hyper
chr17	88791410	88792240	2.79E-27	0.17	0.35	2.06	ENSMUSG00000024107.7	Lhcgr	hyper
chr2	96317666	96318497	8.83E-15	0.17	0.35	2.06	ENSMUSG00000050587.14	Lrrc4c	hyper
chr2	96317671	96318497	1.21E-14	0.17	0.35	2.06	ENSMUSG00000050587.14	Lrrc4c	hyper
chr11	96291207	96291597	2.29E-10	0.16	0.33	2.06	ENSMUSG00000000690.5	Hoxb6	hyper
chr13	104817693	104818748	2.29E-16	0.16	0.33	2.06	ENSMUSG00000021715.12	Cwc27	hyper
chr15	66560765	66561119	1.18E-09	0.16	0.33	2.06	ENSMUSG00000036944.6	Tmem71	hyper
chr5	103210824	103211506	9.41E-09	0.16	0.33	2.06	ENSMUSG00000046709.18	Mapk10	hyper
chr2	177896895	177897197	1.29E-03	0.3	0.62	2.07	ENSMUSG00000074521.9	Gm14327	hyper
chr9	44234809	44235018	3.41E-06	0.15	0.31	2.07	ENSMUSG00000034342.9	Cbl	hyper
chr17	36942291	36942733	2.80E-07	0.14	0.29	2.07	ENSMUSG00000036492.12	Rnf39	hyper
chr5	120887633	120887908	8.99E-05	0.14	0.29	2.07	ENSMUSG00000066861.14	Oas1g	hyper
chr9	108338747	108338950	7.67E-05	0.14	0.29	2.07	ENSMUSG00000063856.7	Gpx1	hyper
chr15	100467123	100467375	6.55E-07	0.27	0.56	2.07	ENSMUSG00000037353.9	Letmd1	hyper
chr5	8998085	8998356	7.84E-04	0.13	0.27	2.08	ENSMUSG00000003623.4	Crot	hyper
chr7	62420008	62420259	9.12E-10	0.13	0.27	2.08	ENSMUSG00000070527.3	Mkrn3	hyper
chr19	41982744	41983011	1.46E-03	0.12	0.25	2.08	ENSMUSG00000025159.8	Mms19	hyper

chr7	102619578	102619854	9.28E-08	0.24	0.5	2.08	ENSMUSG00000073972.2	Olfr553	hyper
chr8	9976923	9977382	1.84E-10	0.12	0.25	2.08	ENSMUSG00000040396.12	Abhd13	hyper
chr7	27688318	27688548	1.28E-12	0.22	0.46	2.09	ENSMUSG00000057093.14	Zfp607b	hyper
chr12	35536091	35536665	1.25E-20	0.42	0.88	2.10	ENSMUSG00000019256.17	Ahr	hyper
chr12	116404780	116405071	5.73E-13	0.21	0.44	2.10	ENSMUSG00000042029.7	Ncapg2	hyper
chr7	98146123	98146384	1.15E-08	0.31	0.65	2.10	ENSMUSG00000074006.3	Omp	hyper
chr4	117124667	117125152	4.73E-11	0.18	0.38	2.11	ENSMUSG00000047671.8	Tctex1d4	hyper
chr14	102983327	102983747	1.13E-08	0.17	0.36	2.12	ENSMUSG00000098557.1	Kctd12	hyper
chr17	88791684	88792329	1.51E-28	0.17	0.36	2.12	ENSMUSG00000024107.7	Lhcgr	hyper
chr18	61726924	61727516	5.02E-11	0.17	0.36	2.12	ENSMUSG00000024580.8	Grpel2	hyper
chr15	54744091	54744369	1.79E-21	0.41	0.87	2.12	ENSMUSG00000037362.8	Ccn3	hyper
chr12	110684612	110685047	2.52E-14	0.32	0.68	2.13	ENSMUSG00000056508.6	1700001K19Rik	hyper
chr16	32165571	32165903	5.45E-08	0.16	0.34	2.13	ENSMUSG00000052384.14	Nrros	hyper
chr15	103241674	103242260	5.14E-06	0.15	0.32	2.13	ENSMUSG00000009575.14	Cbx5	hyper
chr9	82829176	82829786	1.44E-13	0.15	0.32	2.13	ENSMUSG00000032251.12	Irak1bp1	hyper
chr7	98440401	98440617	2.97E-05	0.14	0.3	2.14	ENSMUSG00000074003.4	Gucy2d	hyper
chr17	84682767	84683083	9.81E-09	0.13	0.28	2.15	ENSMUSG00000040505.13	Abcg5	hyper
chr2	90744884	90745296	2.85E-11	0.19	0.41	2.16	ENSMUSG00000008200.15	Fnbp4	hyper
chr3	122292470	122292764	4.81E-07	0.12	0.26	2.17	ENSMUSG00000028121.8	Bcar3	hyper
chr2	19198562	19198842	1.51E-05	0.23	0.5	2.17	ENSMUSG00000037683.14	Armc3	hyper
chr7	104027467	104027982	4.66E-13	0.4	0.87	2.18	ENSMUSG00000066262.4	Olfr640	hyper
chr8	14880198	14880428	1.46E-05	0.11	0.24	2.18	ENSMUSG00000026317.7	Cln8	hyper
chr4	135946086	135946397	1.35E-05	0.1	0.22	2.20	ENSMUSG00000028672.13	Hmgcl	hyper
chr15	95789719	95790139	3.15E-09	0.23	0.51	2.22	ENSMUSG00000064210.7	Ano6	hyper
chr12	105031192	105031401	8.27E-05	0.09	0.2	2.22	ENSMUSG00000021102.4	Glrx5	hyper
chr5	106963624	106964161	8.08E-09	0.09	0.2	2.22	ENSMUSG00000029283.17	Cdc7	hyper
chr19	4046203	4046686	7.73E-14	0.17	0.38	2.24	ENSMUSG00000038155.11	Gstp2	hyper
chr1	171417652	171417989	7.41E-12	0.2	0.45	2.25	ENSMUSG00000103711.1	Tstd1	hyper
chr4	117124742	117125254	1.08E-10	0.16	0.36	2.25	ENSMUSG00000047671.8	Tctex1d4	hyper
chr11	93994453	93995390	2.99E-10	0.12	0.27	2.25	ENSMUSG00000020859.16	Spag9	hyper
chr16	30283704	30284119	5.14E-06	0.12	0.27	2.25	ENSMUSG00000052316.9	Lrrc15	hyper
chr7	7374462	7374824	1.49E-13	0.15	0.34	2.27	ENSMUSG00000110139.1	Gm45783	hyper
chr19	44028988	44029259	3.78E-11	0.29	0.66	2.28	ENSMUSG00000025197.9	Cyp2c23	hyper
chr17	56628036	56628249	7.28E-07	0.14	0.32	2.29	ENSMUSG00000041168.10	Lonp1	hyper
chr17	56628036	56628249	7.28E-07	0.14	0.32	2.29	ENSMUSG00000040828.10	Catsperd	hyper
chr19	4046240	4046686	1.48E-14	0.17	0.4	2.35	ENSMUSG00000038155.11	Gstp2	hyper
chr17	43359902	43360279	5.33E-05	0.13	0.31	2.38	ENSMUSG00000056492.6	Adgrf5	hyper
chr15	95789921	95790259	3.78E-07	0.18	0.43	2.39	ENSMUSG00000064210.7	Ano6	hyper
chr17	84682781	84683083	2.38E-09	0.12	0.29	2.42	ENSMUSG00000040505.13	Abcg5	hyper
chr19	44201427	44201689	1.99E-19	0.31	0.77	2.48	ENSMUSG00000025202.8	Scd3	hyper
chr11	104607147	104607369	8.54E-06	0.16	0.4	2.50	ENSMUSG00000020689.4	Itgb3	hyper
chr17	36942414	36942733	4.75E-07	0.1	0.25	2.50	ENSMUSG00000036492.12	Rnf39	hyper
chr18	36793267	36793743	9.75E-06	0.08	0.2	2.50	ENSMUSG00000001383.9	Zmat2	hyper
chr7	30553480	30553764	1.73E-04	0.08	0.2	2.50	ENSMUSG00000036864.16	Proser3	hyper
chr2	90744919	90745296	9.60E-12	0.14	0.36	2.57	ENSMUSG00000008200.15	Fnbp4	hyper
chr15	11907729	11908184	1.22E-09	0.1	0.26	2.60	ENSMUSG00000022206.7	Npr3	hyper
chr15	81859251	81859842	4.21E-25	0.11	0.29	2.64	ENSMUSG00000048546.8	Tob2	hyper
chr8	95350967	95352150	1.39E-49	0.15	0.4	2.67	ENSMUSG00000031790.8	Mmp15	hyper
chr8	104589456	104589753	7.77E-10	0.27	0.74	2.74	ENSMUSG00000048371.8	Pdp2	hyper
chr1	9544527	9545030	4.12E-08	0.08	0.22	2.75	ENSMUSG00000061024.8	Rrs1	hyper
chr1	185203135	185203824	1.44E-22	0.12	0.33	2.75	ENSMUSG00000039318.12	Rab3gap2	hyper
chr3	19163717	19164163	2.24E-08	0.09	0.26	2.89	ENSMUSG00000027599.9	Armc1	hyper
chr17	83849663	83849956	1.37E-10	0.28	0.83	2.96	ENSMUSG00000000673.9	Haao	hyper
chr19	47854231	47854867	8.84E-10	0.08	0.24	3.00	ENSMUSG00000025068.8	Gsto1	hyper
chr12	112517275	112517655	1.58E-09	0.09	0.28	3.11	ENSMUSG00000054013.6	Tmem179	hyper
chr11	62248061	62248356	2.22E-06	0.08	0.26	3.25	ENSMUSG00000018500.2	Adora2b	hyper
chr5	137601669	137602086	1.58E-21	0.07	0.24	3.43	ENSMUSG00000037221.13	Mospd3	hyper
chr2	38927370	38927777	4.48E-16	0.11	0.39	3.55	ENSMUSG00000063972.13	Nr6a1	hyper
chr11	99024326	99024678	4.66E-09	0.06	0.22	3.67	ENSMUSG00000020914.17	Top2a	hyper
chr9	82829384	82829786	1.65E-14	0.05	0.21	4.20	ENSMUSG00000032251.12	Irak1bp1	hyper
chr7	7278395	7278611	1.34E-20	0.09	0.38	4.22	ENSMUSG00000110105.1	Gm45844	hyper
chr8	116994009	116994310	7.42E-08	0.09	0.44	4.89	ENSMUSG00000034424.5	Gcsh	hyper
chr7	7209544	7210016	1.00E-48	0.05	0.34	6.80	ENSMUSG00000066838.7	Zfp772	hyper
chr11	48825633	48826399	1.57E-49	0.03	0.22	7.33	ENSMUSG00000040350.16	Trim7	hyper
chr5	136692186	136692469	4.67E-11	0.02	0.59	29.50	ENSMUSG00000005474.9	Myl10	hyper

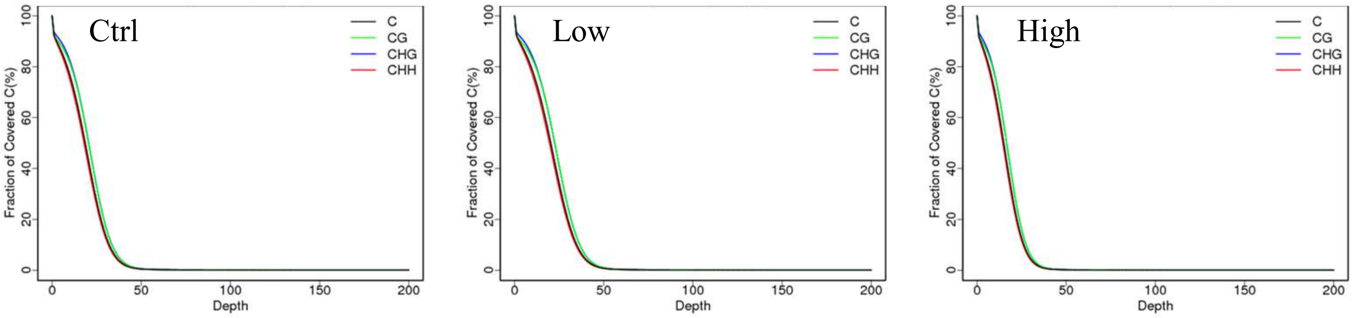

Supplementary Fig S1. Cumulative coverage of corresponding depth in WGBS of livers of fetuses exposed to low (0.3 µg/g bw) and high-dose (3 µg/g bw) PFOS *in-utero*.

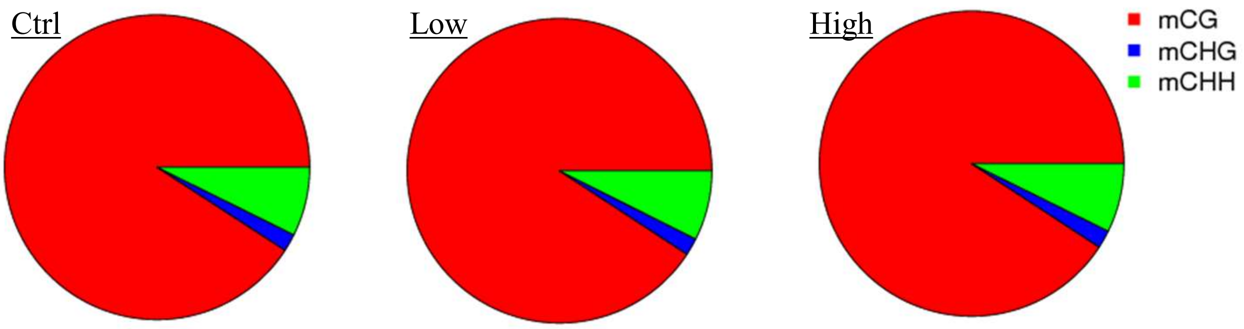

Supplementary Fig S2. The proportion of different types of methylated cytosine in WGBS of livers of fetuses exposed to low (0.3 µg/g bw) and high-dose (3 µg/g bw) PFOS *in-utero*.

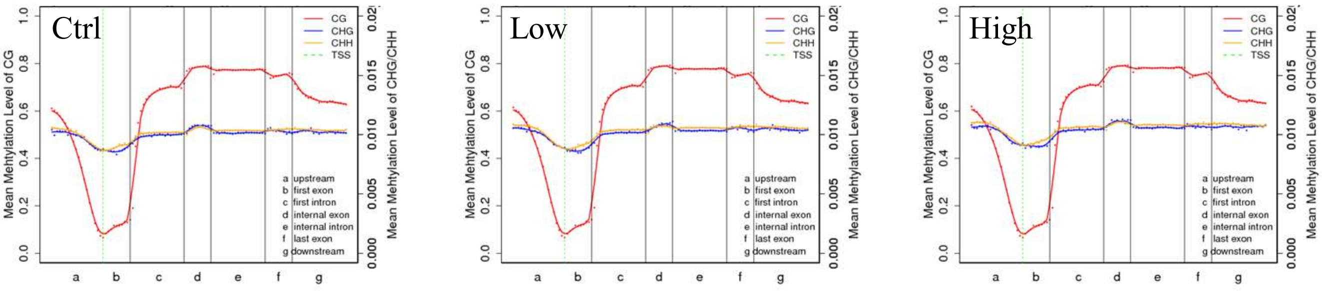

Supplementary Fig S3. Methylation trend in gene regions in WGBS of livers of fetuses exposed to low (0.3 µg/g bw) and high-dose (3 µg/g bw) PFOS *in-utero*.

(A) Hypo-methylated genes

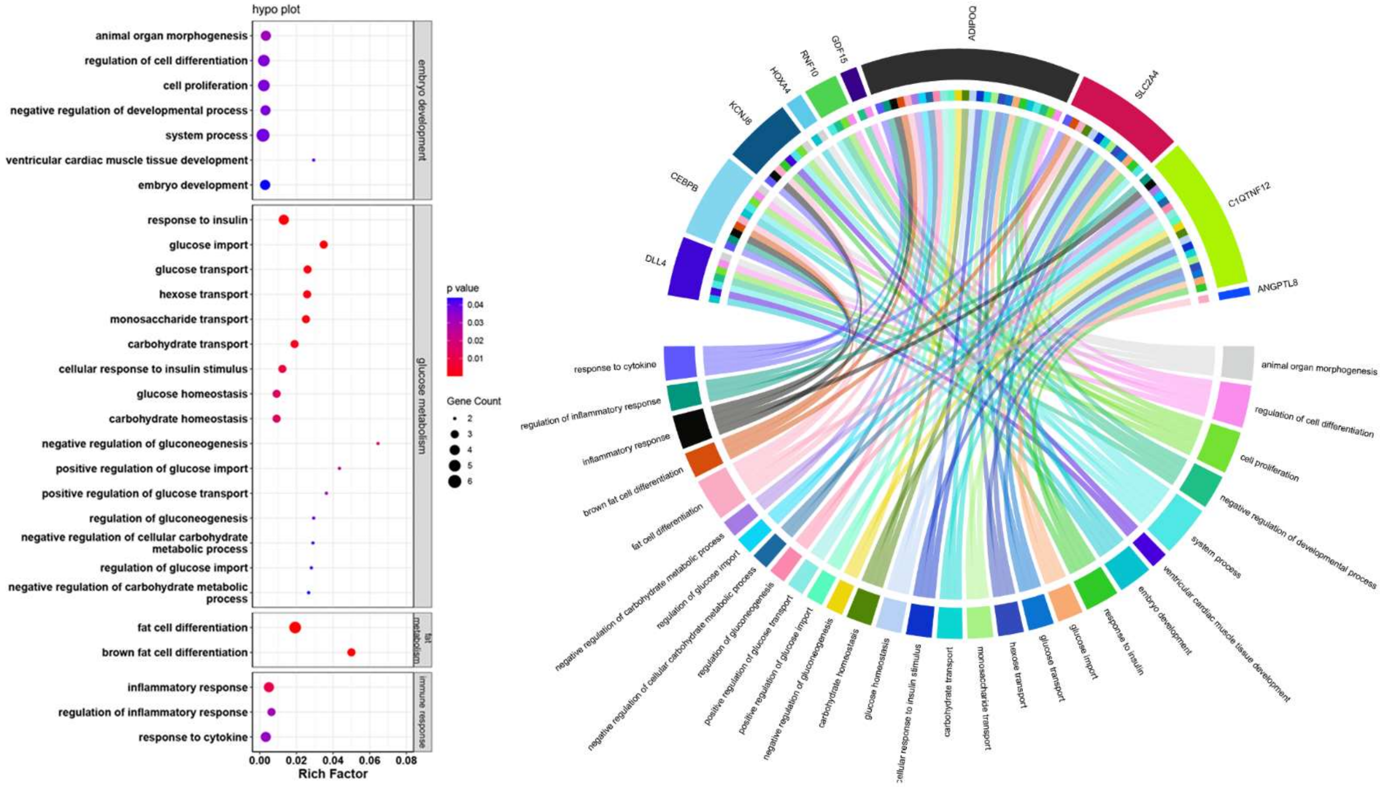

(B) Hyper-methylated genes

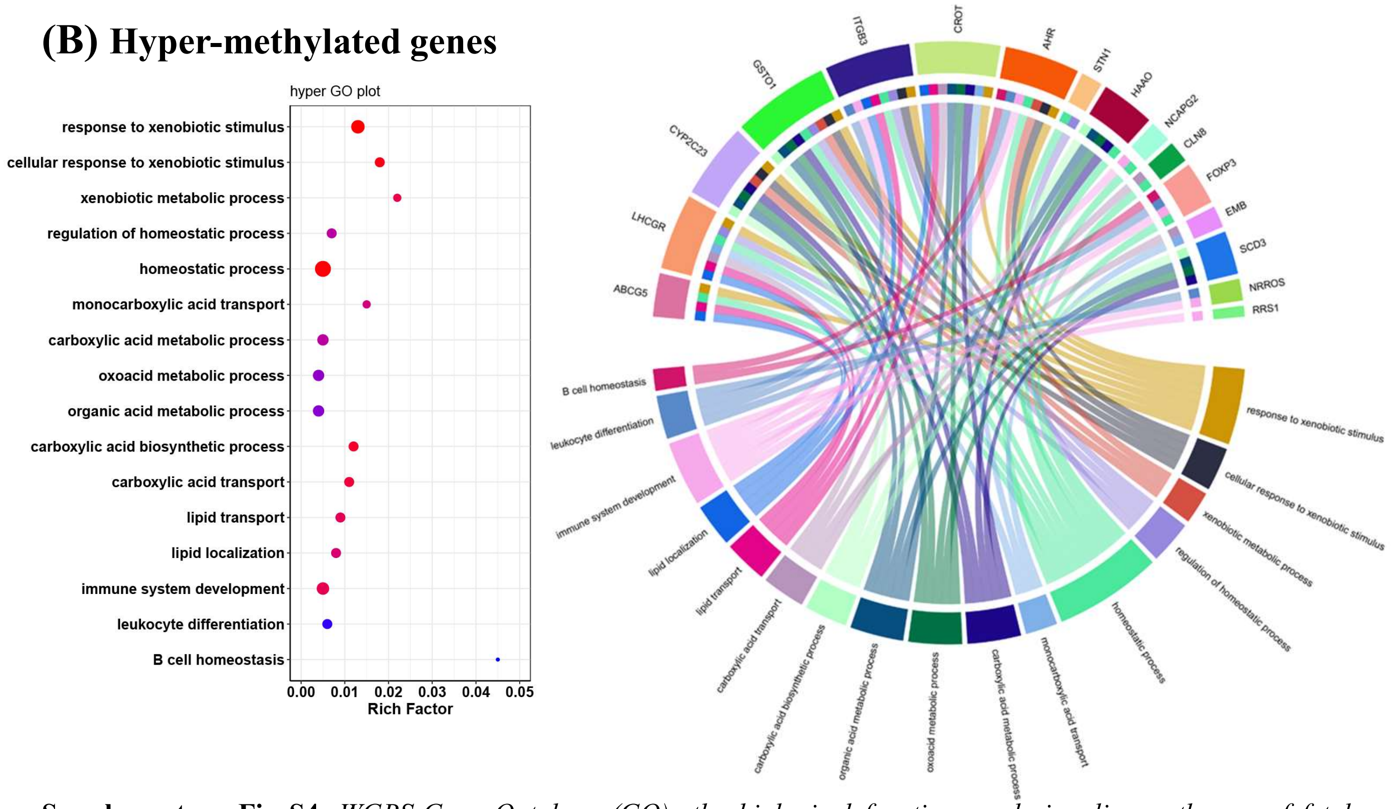

Supplementary Fig S4. *WGBS-Gene Ontology (GO): the biological functions and signaling pathways of fetal hepatic genes, commonly identified in both low- and high-dose PFOS-exposed groups.* (A) An enrichment analysis of hypo-methylated genes, highlighted the involvement of glucose metabolism and inflammatory responses. The size of bubble represented the number of DMR genes. The color of bubble represented the significance of the processes. The right panel: the circos plot showed the relationships and interactions of DMR genes in the highlighted biological processes. (B) An enrichment analysis of hyper-methylated genes, highlighted the involvement of lipid metabolism and xenobiotic responses.

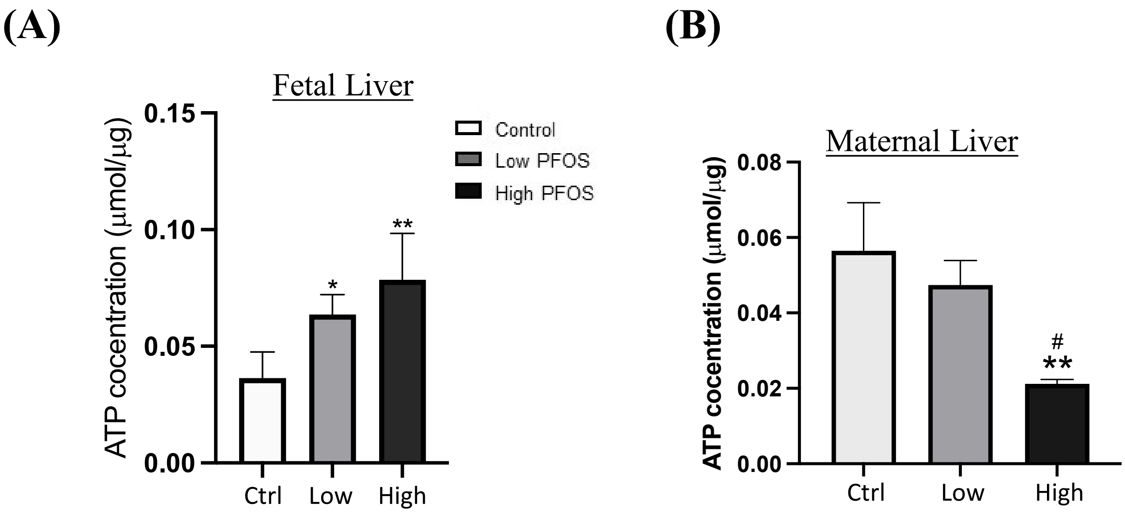

Supplementary Fig S5. (A) Fetal and **(B)** Maternal hepatic ATP levels at gestational day 17.5. Data were presented as the mean ± S.D. *P (treatment vs. control), # P (low-dose vs. high-dose) < 0.05; **P denote <0.01.

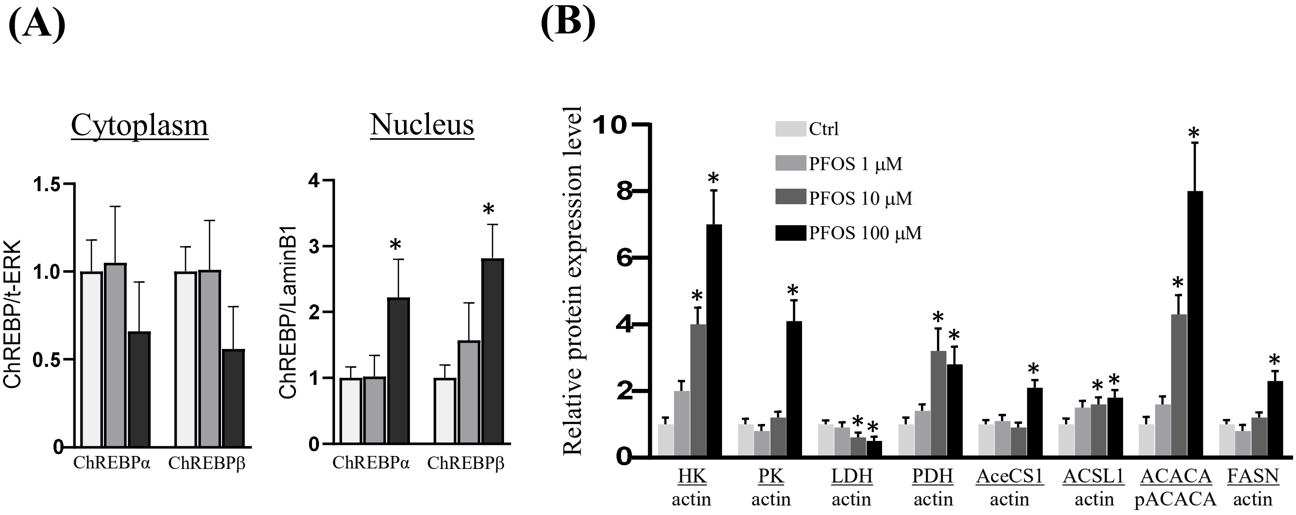

Supplementary Fig S6. A statistical analysis of western blot data from MIHA cells treated with PFOS. **(A)** Cytosolic (left) and nuclear (right) fractions of ChREBPα and ChREBPβ; the protein levels were normalized with total-ERK and laminB1, respectively. **(B)** The protein expression levels of metabolic enzymes for glycolysis and fatty acid synthesis. Data were presented as the mean ± S.D. *P (treatment vs. control) < 0.05. HK: hexose kinase, PK: pyruvate kinase, LDH: lactate dehydrogenase, PDH: pyruvate dehydrogenase, AceCS1: cytoplasmic acetyl-CoA synthetase, ACSL1: acyl-CoA synthetase long chain family member 1, ACACA: acetyl-CoA carboxylase, FASN: fatty acid synthase.
